# Platinum(II) Complexes with Carbene Pincer Chelates for Blue Hyperphosphorescent Organic Light-Emitting Diodes

**DOI:** 10.1021/acs.inorgchem.6c01873

**Published:** 2026-06-22

**Authors:** Guowei Ni, Yufeng Sang, Lin Cheng, Wei He, Shek-Man Yiu, Kai Chung Lau, Guodan Wei, Yun Chi

**Affiliations:** † Department of Materials Science and Engineering, and Center of Super-Diamond and Advanced Films (COSDAF), City University of Hong Kong, Kowloon, Hong Kong SAR 999077, China; ‡ Department of Chemistry, City University of Hong Kong, Hong Kong SAR, Kowloon 999077, China; § Institute of Materials Research, Tsinghua Shenzhen International Graduate School, Tsinghua University, Shenzhen 518055, China

## Abstract

Pt­(II) complexes featuring dicarbene pincer chelates have emerged as promising phosphors for the fabrication of blue organic light-emitting diodes (OLEDs); however, challenges persist in achieving both high-performance and concentration-independent color chromaticity. Herein, we present a series of Pt­(II) complexes featuring a carbene pincer backbone and *N*-mesityl appendages, together with a complementary chloride and pyrazolate entity. These blue Pt­(II) phosphors, **Pt** ***n***, with *n* = 1–5, exhibit a high photoluminescence quantum yield (PLQY) and accelerated radiative transition rate constant (*k*_r_), while the corresponding chloride-to-pyrazolate substitution afforded the “AgCl”-coordinated products **Pt3Ag** and **Pt4Ag** in the presence of Ag_2_O. The representative phosphorescent OLED (PhOLED) based on Pt­(II) phosphor **Pt2** achieved a max. external quantum efficiency (EQ*E*_max_) of 20.1% and a luminance exceeding 100,000 cd m^–2^, with no excimer emission even at high doping concentrations. Furthermore, upon the addition of the terminal emitter **BCzBN**, the resulting hyper-OLED device maintained high EQE values of 21.6% and 18.3% at 1000 and 10,000 cd m^–2^, respectively. These results validated the pivotal role of N-aryl substitution in carbene pincer chelates for developing Pt­(II) emitters aimed at efficient blue PhOLED devices.

## Graphical abstract

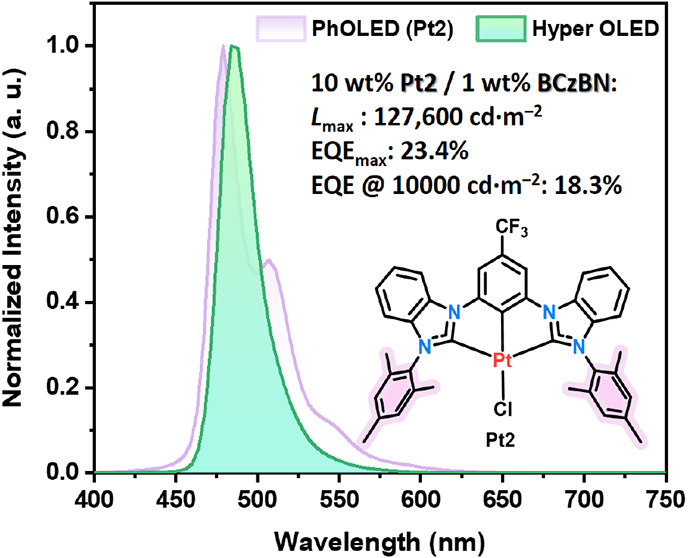

## Introduction

Organic light-emitting diode (OLED) devices have gained significant importance in the fabrication of portable displays and lighting luminaries.^1^ For further maximizing their performance and emission efficiency, it is essential to fully utilize all excitons that are generated during device operation. Hence, fluorescent OLED emitters with a theoretical internal quantum efficiency (IQE) of only 25% were progressively replaced with either phosphors or thermally activated delayed fluorescent (TADF) emitters, both of which can completely harvest singlet and triplet excitons in achieving a maximum IQE of up to 100%. Over the past two decades, in addition to the work on Ir­(III)-based phosphorescent OLED devices, research on Pt­(II) analogues has also grown astonishingly intensive.^2–10^ This is evidenced by the debut of tetradentate Pt­(II) complexes featuring at least one carbene ancillary ligand, which has pushed forward the overall emission efficiency, CIE chromaticity, and operation stability of blue phosphors and associated OLED devices.^11–19^ These results highlighted the importance of the carbene entity in the assembly of Pt­(II)-based phosphors.^20–23^

On the other hand, compared to chelating N-heteroaromatic cyclometalates such as phenylpyridine (ppy) and 1,3-di­(pyridin-2-yl)­benzene (dpb), emissive transition-metal complexes with carbene entities such as phenyl-imidazolylidene (**pim**) and 1,3-di­(imidazolylidene)­benzene (**dimb**) are scarce (Scheme 1), particularly those with N-aryl substituents (i.e., R = N–Ar). Moreover, both the crystal-field strength and π*-orbital energy level of carbene cyclometalates are greater than those of their N-aromatic counterparts.^24^ These properties effectively enlarge the respective S_0_-T_1_ gap and destabilize the metal-centered (MC) *dd* quenching states from their lowest energy T_1_ excited state, making the carbene-based metal complexes display more efficient emission and blue-shifted wavelengths. These are the fundamental reasons that carbene cyclometalates derived from **pim** and **dimb** chelates and their analogues have been extensively tested for transition-metal phosphors, including Pt­(II)-based emitters.

**Scheme 1:**
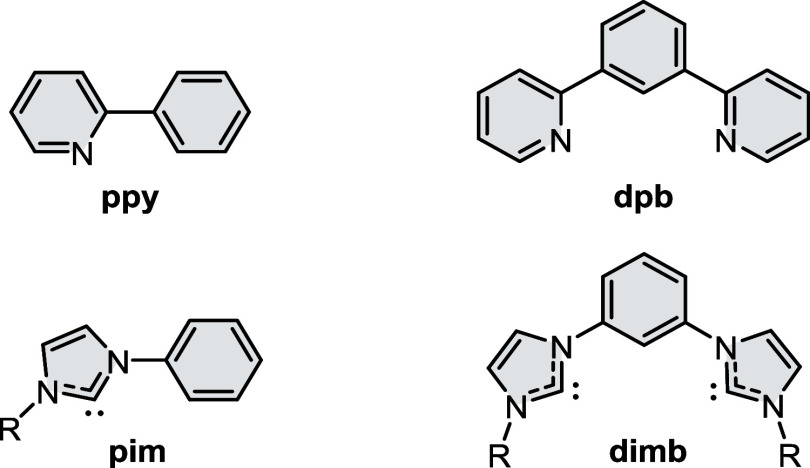
Representative Examples of Bi- and Tridentate Chelates with N-Aromatic Donors and Carbene-Based C-Donors (R = Alkyl or Aryl)

In the present investigation, we aimed to expand the technical scope of Pt­(II) emitters featuring carbene pincer chelates, such as **dimb** and its analogues. Several literature precedents are depicted in Scheme 2, with the employment of tridentate 1,3-di­(imidazolylidene)­benzene or 1,3-di­(benzo­[*d*]­imidazolylidene)­benzene chelates. Also, they are functionalized with N-substituted alkyl appendages (R), such as methyl, *n*-butyl, −CH_2_(TMS), etc. Traditionally, these Pt­(II) pincer complexes were synthesized via a *trans*-metalation procedure with the first addition of Zr­(NMe_2_)_4_,^25^ followed by the exchange with the Pt­(II) metal reagent [Pt­(COD)­Cl_2_].^26^ This operation afforded Pt­(II) complexes **Pt** ^**Me**^ **Cl**, **Pt** ^**Bu**^ **Cl**, **Pt** ^**TMS**^ **Cl**, **Pt** ^**Ar**^ **Cl**, and **Pt** ^**dbib**^ **Cl** in satisfactory yields,^27–29^ with R substituents depicted as superscripts. Notably, **Pt-16** (or **Pt** ^**Me**^ **Cl**) was isolated in poor yield by treating carbene pro-chelate with Ag_2_O in acetonitrile, followed by the addition of K_2_PtCl_4_,^30^ or directly from a mixture of Ag_2_O and K_2_PtCl_4_ in DMSO and heating.^31^ A similar synthetic approach was used in the preparation of **Pt** ^**CN**^ **Cl** with improved yields.^32^ Concurrently, their analogues, namely, **MeCF3Pt**, **IPrCF3Pt**, and **IPr** ***t*** **BuPt**, were synthesized using K_2_CO_3_ as a promoter in refluxing propionic acid, which represents a major improvement with simplified methodologies and more accessible reagents.^33^ Meanwhile, blue phosphorescent OLED and hyperphosphorescent devices with external quantum efficiency (EQE) of up to 33.59%, and chromaticity coordinates CIE_*xy*_ of (0.12, 0.15) were obtained with **IPr** ***t*** **BuPt** as a dopant or sensitizer, and **ν-DABNA** as a terminal emitter. Overall, these studies showed that Pt­(II) complexes featuring pincer carbene chelates can be of great value in the fabrication of blue OLED devices with high performance. Inspired by these findings, we decided to further functionalize the Pt­(II) emitters by employing bulky N-aryl substituents on the pincer carbene and replacing the chloride ligand with functional pyrazolate to fine-tune their chemical and photophysical characteristics and respective device performances. Our results on emitter synthesis and device characterizations are presented below.

**Scheme 2:**
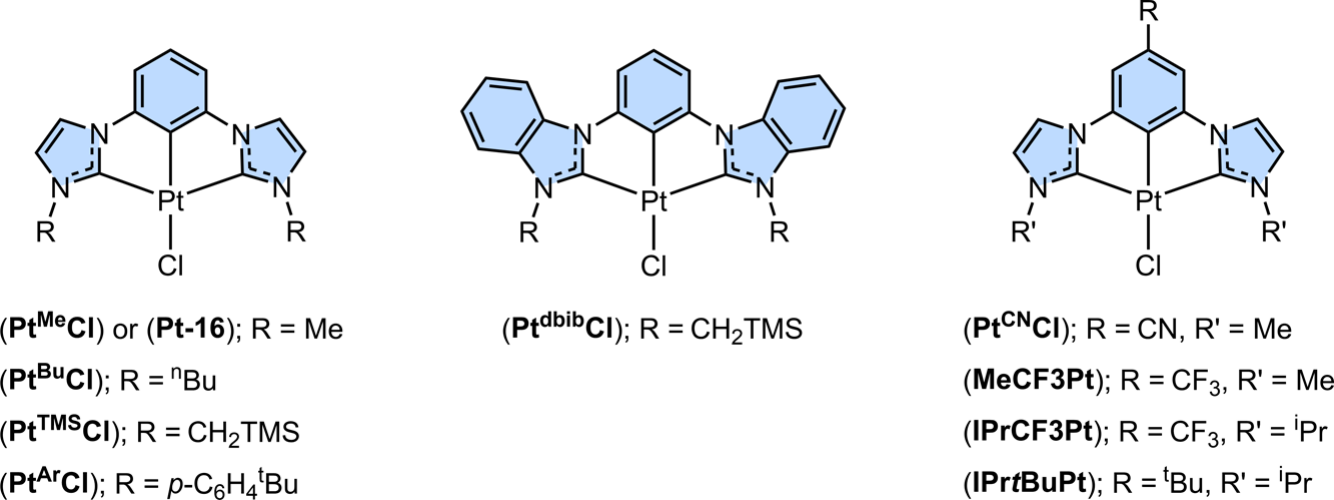
Drawings of the Pincer Carbene-Based Pt­(II) Complexes Reported in the Literature

## Results and Discussion

### Synthesis and Characterizations

Our endeavor started with the preparation of prochelates 3,3′-(1,3-phenylene)­bis­(1-mesityl-benzo­[*d*]­imidazol-3-ium) with R substituents *tert*-butyl (**C1**) and CF_3_ (**C2**), whose structural drawings and synthetic approaches are depicted in Schemes 3 and S1, respectively. First, *N*-(2-bromophenyl)-2,4,6-trimethylaniline (intermediate **A**) was obtained from 1-bromo-2-iodobenzene and 2,4,6-trimethylaniline in the presence of a palladium catalyst. After that, symmetrical bis­(*N*-mesitylbenzene-1,2-diamine) (**B1** and **B2**) was prepared from direct amination of the respective functional 1,2-dibromobenzene, while cyclization of **B1** and **B2** with trimethyl orthoformate afforded the anticipated pincer prochelates **C1** and **C2**, to which the employed synthetic procedures are analogous to the literature procedures.^34,35^

**Scheme 3:**
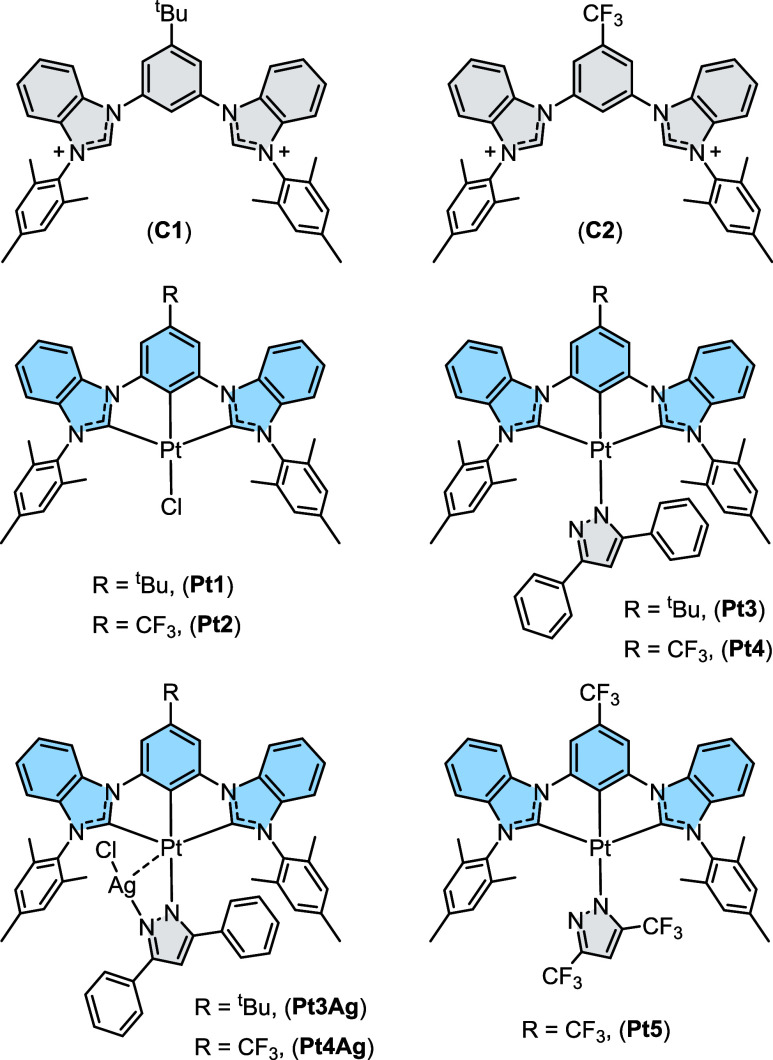
Structural Drawings of Studied Pt­(II) Complexes with Pincer Carbene Chelate

Subsequently, chloride complexes **Pt1** and **Pt2** were prepared by treating prochelates **C1** and **C2** with K_2_PtCl_4_ in the presence of excess K_2_CO_3_ in refluxing propionic acid. The substitution of chloride with 3,5-diphenylpyrazole can be achieved under two distinct conditions. Those with Ag_2_O as the promoter in CH_2_Cl_2_ solution afforded the “AgCl”-coordinated Pt­(II) complexes **Pt3Ag** and **Pt4Ag**, while the application of Na_2_CO_3_ in ethanol afforded the anticipated Pt­(II) complexes **Pt3** and **Pt4** instead. Moreover, the removal of “AgCl” fragment from **Pt3Ag** and **Pt4Ag** can be effectively executed under direct vacuum sublimation, while the addition of the “AgCl” coordination unit can be achieved by sequential treatment of **Pt3** (or **Pt4**) with AgPF_6_ and ^n^Bu_4_NCl in refluxing CH_2_Cl_2_ solution. Lastly, treatment of **Pt2** with electron-deficient 3,5-ditrifluoromethylpyrazole yielded only the Pt­(II) product **Pt5** even in the presence of Ag_2_O, indicating the failure of the 3,5-ditrifluoromethylpyrazolate ligand in supporting “AgCl” coordination. This result confirmed the intrinsic nature of pyrazolate in determining the reactivity pattern within this series of Pt­(II) complexes.

Moreover, single-crystal X-ray structural analyses of **Pt2**, **Pt3**, **Pt3Ag**, **Pt4**, and **Pt5** were performed, and the structural drawings are depicted in Figures 1–5, respectively. The essential metal–ligand bond distances are summarized in Table 1 for deciphering the influence of the coordination ligand. First, all their N-mesityl appendages were perpendicular to the coordination framework of the pincer chelate, which could significantly reduce the intermolecular stacking interactions known for Pt­(II) metal complexes.^36–41^ Moreover, among Pt­(II) complexes **Pt2**, **Pt4**, and **Pt5** featuring **C2** pincer chelate, replacement of chloride with pyrazolates exerted little influence on the Pt–C_(carbene)_ distances, but imposed a relatively larger reduction in the Pt–C_(Ph)_ distance, which could be associated with the inherent electronic properties of the pyrazolate ligand, as shown by the associated variation in the Pt–N_(pz)_ distance. On the other hand, the addition of the “AgCl” unit to **Pt3** in **Pt3Ag** strengthened both the Pt–C_(carbene)_ and Pt–N_(pz)_ interactions, together with a slight lengthening of the Pt–C_(Ph)_ bond distance, which is consistent with the DFT results.

**Figure 1:**
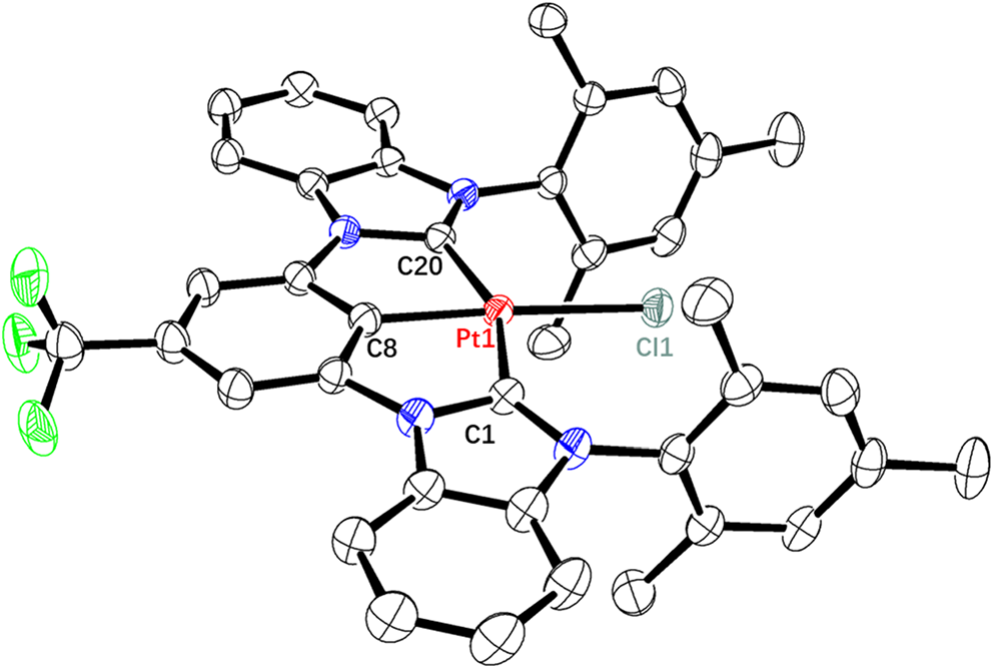
Structural drawing of **Pt2** at the 30% probability level; hydrogen atoms are omitted for clarity. Selected bond distances: Pt1–C1 = 2.024(3), Pt1–C8 = 1.940(3), Pt1–C20 = 2.023(3), and Pt1–Cl1 = 2.360(7) Å; selected bond angles: C1–Pt1–C8 = 79.08(13) and C8–Pt1–C20 = 79.01(12)°.

**2:**
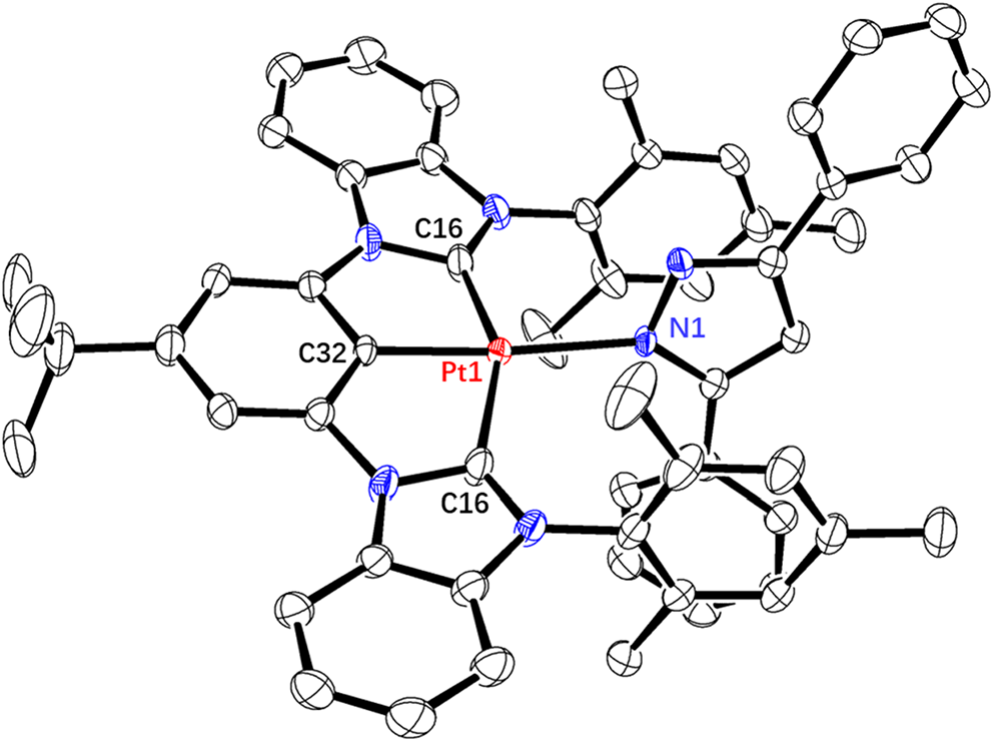
Structural drawing of **Pt3** at the 30% probability level; hydrogen atoms are omitted for clarity. Selected bond distances: Pt1–C16 = 2.056(3), Pt1–C32 = 1.944(5), and Pt1–N1 = 2.126(4) Å; selected bond angles: C16–Pt1–C32 = 77.03(4)°.

**3:**
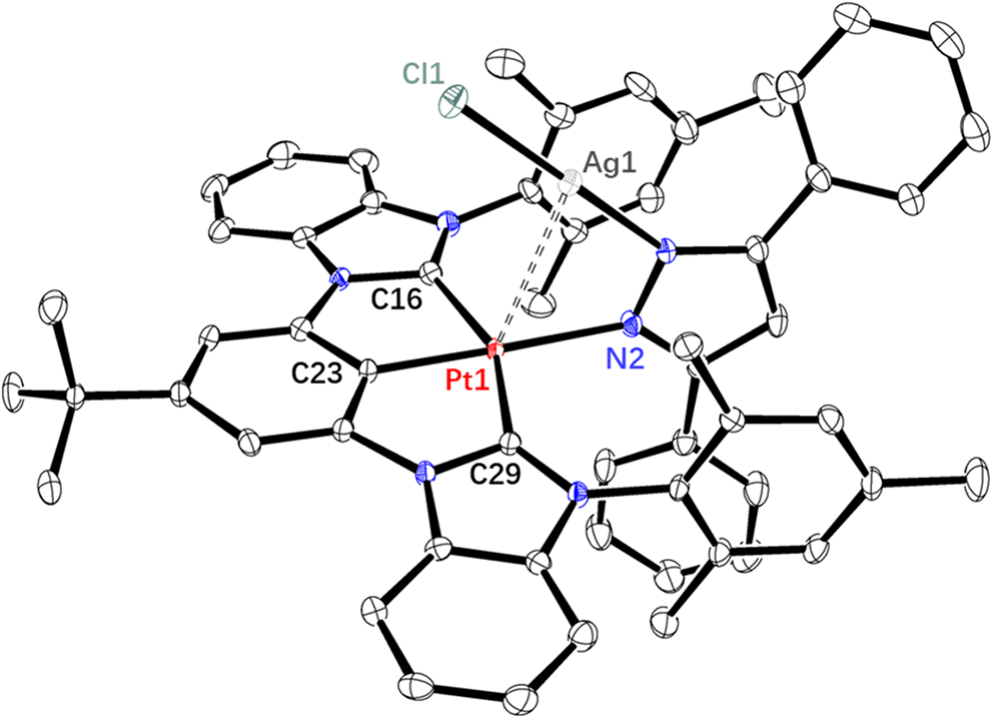
Structural drawing of **Pt3Ag** at the 30% probability level; hydrogen atoms are omitted for clarity. Selected bond distances: Pt1–C16 = 2.032(4), Pt1–C23 = 1.950(3), Pt1–C29 = 2.030(4), Pt1–N2 = 2.114(3), and Pt1–Ag1 = 3.011(3) Å; selected bond angles: C16–Pt1–C23 = 78.28(15) and C23–Pt1–C29 = 78.80(15)°.

**4:**
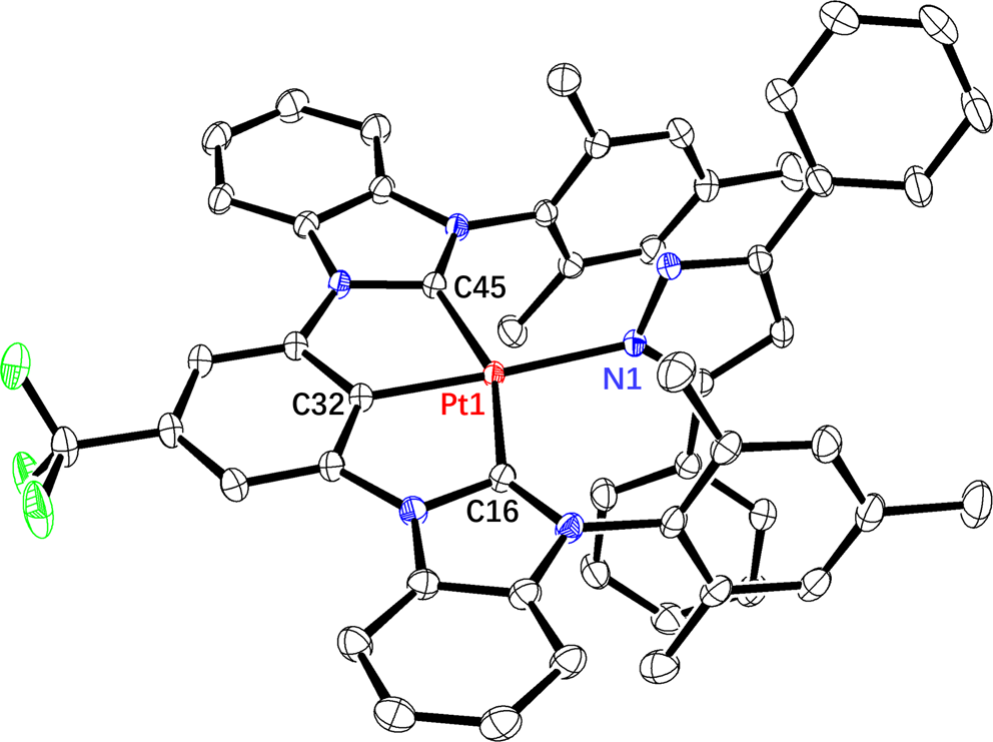
Structural drawing of **Pt4** at the 30% probability level; hydrogen atoms are omitted for clarity. Selected bond distances: Pt1–C16 = 2.021(2), Pt1–C32 = 1.950(2), Pt1–C45 = 2.030(2), and Pt1–N1 = 2.0991(19) Å; selected bond angles: C16–Pt1–C32 = 78.80(10) and C32–Pt1–C45 = 78.99(9)°.

**Figure 5:**
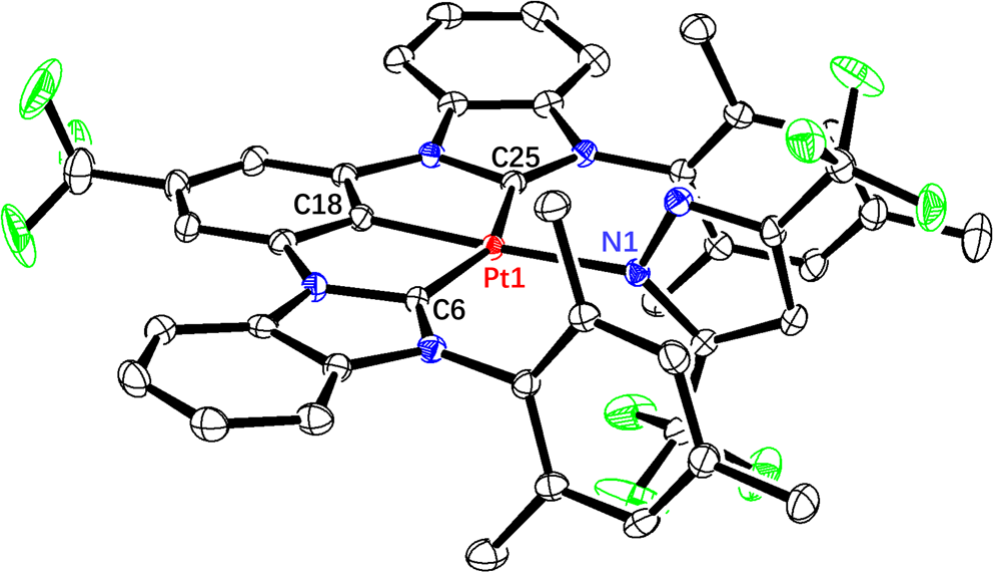
Structural drawing of **Pt5** at the 30% probability level; hydrogen atoms are omitted for clarity. Selected bond distances: Pt1–C25 = 2.026(4), Pt1–C18 = 1.939(4), Pt1–C6 = 2.021(4), and Pt1–N1 = 2.092(3) Å; selected bond angles: C25–Pt1–C18 = 78.70(15), and C18–Pt1–C6 = 79.28(15)°.

**Table 1:** Comparative Pt–Ligand Bond Distances (Å) of the Studied Pt­(II) Complexes

	Pt–C_(Ph)_	Pt–C_(carbene)_	Pt–Cl or Pt–N_(pz)_
**Pt2**	1.940(3)	2.023(3)/2.024(3)	2.360(7)
**Pt3**	1.944(5)	2.056(3)/2.056(3)	2.126(4)
**Pt3Ag**	1.950(3)	2.030(4)/2.032(4)	2.114(3)
**Pt4**	1.950(2)	2.021(2)/2.030(2)	2.099(2)
**Pt5**	1.939(4)	2.021(4)/2.026(4)	2.092(3)

Next, ^195^Pt NMR spectra were recorded for all the prepared Pt­(II) carbene complexes. The chemical shifts of **Pt1** and **Pt2** occurred at δ−3983 and −3924, respectively, and their difference indicated the relative deshielding character of the electron-deficient CF_3_ group vs the electron-donating *t*-butyl substituent on the carbene pincer chelate. Moreover, a comparison of these chemical shift data among pyrazolate complexes **Pt3** (−4057), **Pt4** (−4011), and **Pt5** (−4101) implied a further deshielding effect of the pyrazolate ligand vs chloride, while the high-field shifting became larger with the more electron-deficient 3,5-ditrifluoromethylpyrazolate on **Pt5**. Finally, the “AgCl” units on **Pt3Ag** (−4131) and **Pt4Ag** (−4087) caused a high-field shift like that of the CF_3_ group, reflecting its Lewis acidic nature. Interestingly, no obvious ^195^Pt-^107,109^Ag coupling was detected in our “AgCl” adducts **Pt3Ag** and **Pt4Ag**, despite the direct Pt–Ag interactions, which contrasts with the Pt–Ag clusters reported in the literature.^42^

### Photophysical, Electrochemical, and Thermal Characterizations

The absorption and emission spectra of all Pt­(II) complexes were measured in toluene at RT to confirm their photophysical behaviors. The respective spectra and numerical data are shown in Figure 6a,b and Table 2. As can be seen, the differences in their absorption spectra are minor, with all spectra exhibiting two main transitions, one at ≤ 320 nm and the second at ∼380 nm, the latter flanked by a lower-energy shoulder. These absorption bands are then assigned to the intraligand π–π* transition and mixed ligand-centered π–π* and metal-to-ligand charge transfer (MLCT) processes, respectively. On the other hand, all Pt­(II) complexes showed well-resolved emissions with structured vibronic patterns spanning the 477–572 nm region, which is consistent with the dominant π–π* process at the excited-state manifolds. The high photoluminescence quantum yields (PLQY) of 0.98–0.73 agreed with the high rigidity of the Pt­(II) coordination framework and the much destabilized MC *dd* excited states induced by the high crystal-field strength of the carbene pincer chelate.^32^ Moreover, their negligible change in the emission profile over the concentrations of 1–0.1 × 10^–5^ M, meaning no excimer or exciplex emission, is consistent with the large steric hindrance exerted by two orthogonal N-mesityl appendages on the pincer chelate (Figure S1). According to the observed lifetime (τ_obs_) derived from the transient decay analysis (Figure S2), their radiative rate constants (*k*_r_) of 3.80–2.15 × 10^5^ s^–1^ and nonradiative rate constants (*k*_nr_) of 1.09–0.03 × 10^5^ s^–1^ could be obtained using the equations

$$k_{r}=\mathrm{PLQY}/\tau_{\mathrm{obs}}\;\mathrm{and}\;k_{\mathrm{nr}}=\left(1-\mathrm{PLQY}\right)/\tau_{\mathrm{obs}}$$

The obtained photophysical parameters, such as PLQY, *k*_r_, and *k*_nr_, are relatively better than those of their Pt­(II) precedents bearing other appendages, such as *N*-methyl, N-butyl, and N-TMS, *N*-isopropyl, and on the carbene pincer chelate of the Pt­(II) complexes, as shown in Scheme 2, confirming the advantages of the current designs with N-mesityl appendages.

**Table 2:** Photophysical Data of the Studied Pt­(II) Complexes Recorded in Toluene Solution at RT

	λ_abs_ [nm]^a^	λ_PL_ [nm]^b^	fwhm	PLQY^b^	τ_obs_ [μs]	τ_rad_ [μs]	*k*_r_ [10^5^ s^–1^]^c^	*k*_nr_ [10^5^ s^–1^]^c^
**Pt1**	327 (1.2), 388 (1.0)	497, 531, 570	52 nm	0.98	2.98	3.01	3.32	0.03
**Pt2**	295 (2.4), 326 (0.87), 386 (0.76)	477, 509, 544	41 nm	0.97	2.55	2.63	3.80	0.12
**Pt3**	299 (3.5), 383 (0.59)	495, 521, 558	61 nm	0.91	2.89	3.18	3.15	0.31
**Pt3Ag**	295 (3.3), 386 (0.56)	497, 529, 570	54 nm	0.86	3.16	3.67	2.72	0.44
**Pt4**	290 (3.4), 379 (0.62)	477, 508, 543	80 nm	0.84	2.52	3.00	3.33	0.63
**Pt4Ag**	287 (4.9), 382 (0.51)	477, 509, 545	46 nm	0.73	2.49	3.42	2.93	1.09
**Pt5**	290 (3.5), 328 (1.0), 386 (0.86)	464, 495, 529	45 nm	0.89	4.14	4.65	2.15	0.27

a UV–vis spectra were recorded in toluene at 10^–5^ M at RT, and the extinction coefficient (ε) is given in parentheses with units of 10^4^ M^–1^·cm^–1^.

b Photophysical data recorded in degassed toluene at RT. Coumarin 102 in methanol (PLQY = 87% and λ_max_ = 480 nm) was employed as the standard.

c *k*_r_ and *k*_nr_ represent the radiative and nonradiative rate constants, respectively.

**Figure 6:**
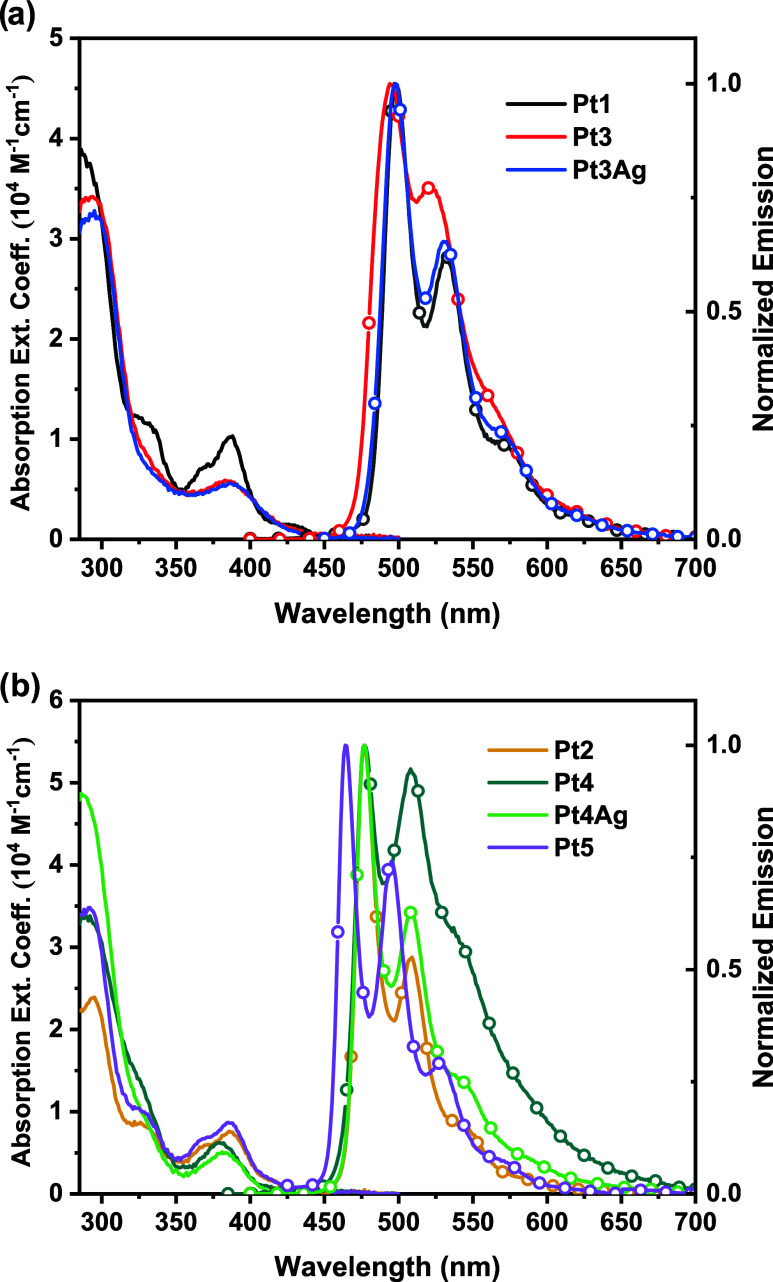
UV–vis absorption and emission spectra of the studied Pt­(II) complexes with (a) carbene pincer chelate **C1** and (b) carbene pincer chelate **C2** recorded in toluene solution at RT.

Emission tuning was then conducted using several structural modifications. For example, the replacement of *t*-butyl with a CF_3_ substituent on the pincer chelate caused a blue shift in their first emission band, that is, from **Pt1** (497 nm) to **Pt2** (477 nm) and from **Pt3** (495 nm) to **Pt4** (477 nm). This result verified the enlargement of the π–π* gap on the pincer chelate. Moreover, the substitution of chloride with 3,5-diphenylpyrazolate induced only a small change in the ligand-centered π–π* transition energy, as shown by their first peak max., that is, from **Pt1** (497 nm) to **Pt3** (495 nm) and from **Pt2** (477 nm) to **Pt4** (477 nm), while the introduction of electron-deficient 3,5-ditrifluoromethylpyrazolate yielded a much greater blue shifting instead, that is, from **Pt2** (477 nm) to **Pt5** (465 nm). This can be understood by the reduction in the MLCT contribution percentage, to which a reduced radiative rate constant (*k*_r_) could serve as another supportive evidence; c.f., *k*_r_ = 3.80 × 10^5^ s^–1^ (**Pt2**) and 2.15 × 10^5^ s^–1^ (**Pt5**). Moreover, the fwhm of **Pt4** (80 nm) is significantly greater than that of **Pt4Ag** (46 nm) and **Pt5** (45 nm) in toluene, which can be attributed to the high polarity induced by the electron-donating 3,5-diphenylpyrazolate fragment to the electron-deficient CF_3_-substituted pincer chelate in **Pt4**. This molecular polarity can be suppressed by either the addition of Lewis acidic “AgCl” to 3,5-diphenylpyrazolate to give **Pt4Ag**, or by replacing the dual phenyl groups on pyrazolate with CF_3_ substituents in **Pt5**, and the fwhm resumed to normal, as shown in Table 2. Finally, the addition of “AgCl” to the Pt­(II) framework caused minor changes in the emission peak wavelength, PLQY, and *k*_r_ values, c.f., from **Pt3** (495 nm, 0.91, 3.15 × 10^5^ s^–1^) to **Pt3Ag** (497 nm, 0.86, 2.72 × 10^5^ s^–1^) and from **Pt4** (477 nm, 0.84, 3.33 × 10^5^ s^–1^) to **Pt4Ag** (477 nm, 0.73, 2.93 × 10^5^ s^–1^). Remarkably, none of these modifications substantially deteriorated the photophysical properties.

Moreover, the emission of Pt­(II) complexes **Pt1**–**Pt5** was also recorded in cast-doped polystyrene thin films at 2 wt %, and both the spectral profile and numeric data are presented in Figure S3 and Table S1, respectively. As can be seen, these data resemble those recorded in toluene solution at high dilution, reconfirming the advantage of *N*-mesityl appendages in improving the photoluminescent characteristics of Pt­(II) complexes without this substituent.^33^

Cyclic voltammetry (CV) and thermogravimetric analyses were also conducted (Table S2 and Figure S4). Irreversible or quasi-reversible oxidation peaks were detected for all Pt­(II) complexes, depending on the relative electron density on the Pt­(II) metal center. The calculated onset potentials can be grouped in accordance to the associated functional groups, as follows: (i) **Pt1** (*E*_ox_ = 0.75 V) < **Pt2** (*E*_ox_ = 1.02 V), (ii) **Pt4** (*E*_ox_ = 0.40 V) < **Pt5** (*E*_ox_ = 0.56 V) < **Pt2** (*E*_ox_ = 1.02 V), (iii) **Pt3** (*E*_ox_ = 0.34 V) < **Pt3Ag** (*E*_ox_ = 0.48 V), and (iv) **Pt4** (*E*_ox_ = 0.40 V) < **Pt4Ag** (*E*_ox_ = 0.51 V). Since this oxidation process mainly occurred at the Pt­(II) metal center, their recorded peak potentials can be used as a gauge for the relative electron density at the metal center. From (i), we conclude that both the chloride on the Pt­(II) metal atom and the CF_3_ substituent of the pincer chelate would reduce the oxidation peak potential. From (ii), we can see the substituent effect of two kinds of pyrazolates vs chloride, and from both (iii) and (iv), we identified the electron-withdrawing nature of the “AgCl” coordination unit. Finally, the HOMO and LUMO levels can be calculated using the oxidation potentials and optical energy gaps obtained from the onset of the absorption band. Thermogravimetric (TG) analysis estimated the decomposition temperature (*T*_d_) of these Pt­(II) complexes to be between 354 and 388 °C, where *T*_d_ indicates the temperature at which a weight loss of 5% occurs (Figure S5).

### Theoretical Investigation

To explore the impact of chelate modifications on the electronic structures and photophysical properties of these Pt­(II) complexes, time-dependent density functional theory (TD-DFT) calculations were conducted,^43–45^ and the electronic transitions associated with the S_1_ and T_1_ states were analyzed. Further computational details are provided in the Supporting Information (SI).

The S_0_ and T_1_ structures of **Pt2**, **Pt3**, **Pt3Ag**, **Pt4**, and **Pt5** were optimized at the B3LYP-D3­(BJ)/def2-SVP level in toluene.^46–52^ The mean absolute deviations (MADs) between the optimized bond distances for the optimized S_0_ structures (Table S3) and the corresponding experimental values (Table 1) are 0.018, 0.019, and 0.039 Å for Pt–C_(carbene)_, Pt–C_(Ph)_, and Pt–N_(pz)_ distances (or Pt–Cl for **Pt2**), respectively, indicating good agreement with the experimental data.

TD-DFT calculations, performed on the optimized S_0_ structures of the studied Pt­(II) complexes in toluene, were used to compute the vertical excitation energies for the S_0_ → S_1_ and S_0_ → T_1_ transitions. For **Pt2**, **Pt3**, **Pt3Ag**, **Pt4**, and **Pt5**, the computed values are 420, 469, 408, 492, and 369 nm, respectively, which align with the experimentally observed absorption tails around 400∼450 nm (Figure 6 and Table 3). For the S_0_ → T_1_ transition, the vertical excitation energies for **Pt2**, **Pt3**, **Pt3Ag**, **Pt4**, and **Pt5** are 462, 479, 451, 497, and 433 nm, respectively. These values yield an MAD of 0.14 eV from the experimental emission peak max. λ_max_ (477, 495, 497, 477, and 464 nm, Table 2). The adiabatic emission energies of the T_1_ → S_0_ transitions (465, 478, 469, 496, and 446 nm for **Pt2**, **Pt3**, **Pt3Ag**, **Pt4**, and **Pt5**, respectively) further improve the agreement, with a smaller MAD of 0.10 eV.

Natural transition orbital (NTO) analysis was conducted based on the optimized S_0_ structures to gain deeper insight into the S_0_ → T_1_ transitions in the studied Pt­(II) complexes (Figure 7).^53^ For all the studied Pt­(II) complexes, the virtual NTOs are primarily localized on the carbene pincer chelates (in π* orbitals). The occupied NTOs, however, exhibit complex-dependent distributions: in **Pt2**, the occupied NTOs reside predominantly on the Pt­(II) metal center, carbene chelates, and chloride (in π orbitals); in **Pt3** and **Pt4**, on the pyrazolate (in π orbitals); in **Pt3Ag** and **Pt5**, on the Pt­(II) metal center and carbene chelates (in π orbitals). Consequently, **Pt2** exhibits a combination of metal-to-ligand charge transfer (MLCT) and ligand-to-ligand charge transfer (LLCT, i.e., interligand charge transfer between the carbene pincer chelates and chloride) characteristics. **Pt3** and **Pt4** show predominantly LLCT (i.e., interligand charge transfer between the carbene pincers and pyrazolate) characteristics. **Pt3Ag** and **Pt5** exhibit mixed MLCT, LLCT (i.e., interligand charge transfer between the carbene pincers and pyrazolate), and LC characteristics.

**Figure 7:**
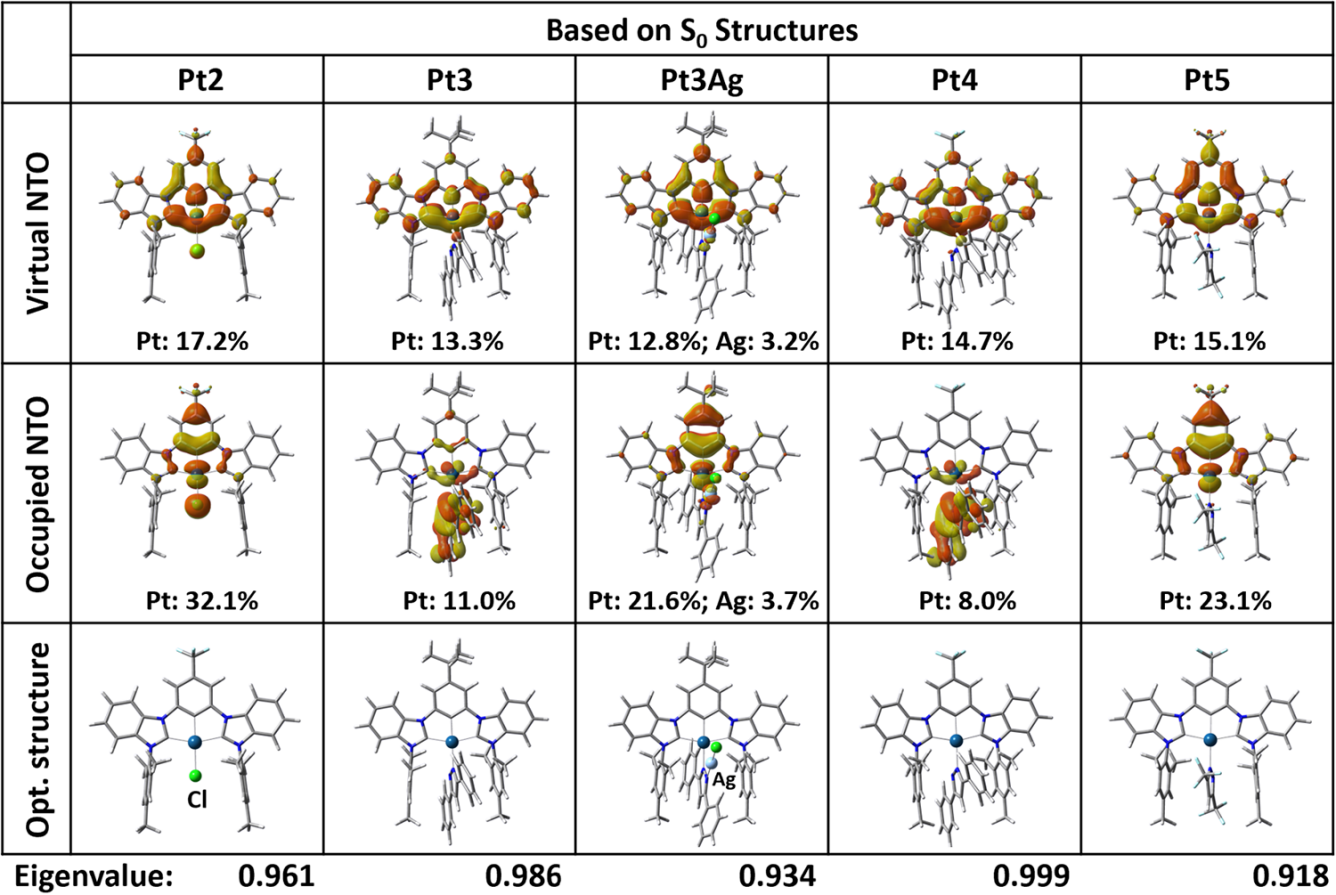
Dominant eigenvalues and NTO pairs for the S_0_ → T_1_ excitation of the studied complexes at their optimized S_0_ structures in toluene, including the contributions of the Pt­(II) center and Ag atom to the NTOs.

To quantitatively evaluate the contributions of MLCT, ligand-to-metal charge transfer (LMCT), LLCT, LC, and metal-centered (MC) characters to the S_0_ → T_1_ transition for all studied Pt­(II) complexes, the interfragment charge transfer (IFCT) analysis was performed using Multiwfn software based on their optimized S_0_ structures (Table 3).^54,55^ For **Pt2**, the MLCT and LLCT contributions are 25.9% and 42.1%, respectively. **Pt3Ag** and **Pt5** show mixed contributions: MLCT (17.6% and 18.7%), LLCT (50.3% and 40.3%), and LC (19.8% and 26.7%). **Pt3** and **Pt4** exhibit dominant LLCT characteristics (70.1% and 73.0%). All results are consistent with the NTO analysis. Interestingly, for the species exhibiting a positive net MLCT characteristics (**Pt2**, **Pt3Ag**, and **Pt5**, in Table 3), the calculated net MLCT percentages (14.4%, 7.7%, and 7.4%) show a positive correlation with the radiative rate constants *k*_r_ (3.80 × 10^5^, 2.72 × 10^5^, 2.15 × 10^5^ s^–1^). For the **Pt3** and **Pt4** complexes with a negative net MLCT, the *k*_r_ values (3.15 × 10^5^ and 3.33 × 10^5^ s^–1^, Table 2) are more closely aligned with their LLCT percentages (70.1% and 73.0%).

**Table 3:** TD-DFT Calculation Results and Molecular Orbital (MO) Contributions (>15%) of the S_0_ → S_1_ and S_0_ → T_1_ Excitations, and the Charge Transfer Characteristic Assignments for the S_0_ → T_1_ Excitation of the Studied Complexes (at the Optimized S_0_ Structure) in Toluene

						assignment^c^					
	*E*_HOMO_/*E*_LUMO_ ^a^ (eV)	excitation	λ^b^ [nm/eV]	*f* b	MO contribution^b^ (>15%)	MLCT	LMCT	LLCT	LC	MC	net MLCT^d^
**Pt2**	–5.63/–1.85	S_0_ → T_1_	462/2.68	0.0000	HOMO–1 → LUMO (84.7%)	25.9%	11.4%	42.1%	15.5%	5.1%	14.4%
		S_0_ → S_1_	420/2.95	0.0001	HOMO → LUMO (98.0%)						
**Pt3**	–4.97/–1.74	S_0_ → T_1_	479/2.59	0.0000	HOMO → LUMO (90.3%)	9.4%	11.6%	70.1%	7.5%	1.4%	–2.2%
		S_0_ → S_1_	469/2.65	0.0108	HOMO → LUMO (98.3%)						
**Pt3Ag**	–5.56/–1.89	S_0_ → T_1_	451/2.75	0.0000	HOMO–1 → LUMO (56.8%)	17.6%	9.9%	50.3%	19.8%	2.5%	7.7%
					HOMO–3 → LUMO (23.1%)						
		S_0_ → S_1_	408/3.04	0.0046	HOMO → LUMO (96.6%)						
**Pt4**	–5.05/–1.94	S_0_ → T_1_	497/2.49	0.0000	HOMO → LUMO (97.5%)	6.8%	13.1%	73.0%	5.9%	1.1%	–6.3%
		S_0_ → S_1_	492/2.52	0.0010	HOMO → LUMO (98.8%)						
**Pt5**	–6.09/–2.01	S_0_ → T_1_	433/2.87	0.0000	HOMO–1 → LUMO (55.4%)	18.7%	11.2%	40.3%	26.7%	3.1%	7.4%
					HOMO → LUMO (24.3%)						
		S_0_ → S_1_	369/3.36	0.0031	HOMO → LUMO (95.6%)						

a The *E*_HOMO_ and *E*_LUMO_ of the optimized S_0_ structures in the gas phase were calculated at the B3LYP-D3­(BJ)/def2-SVP level.

b Vertical excitation energy (λ), oscillator strength (*f*), and MO contribution were obtained by the TD-DFT method at the B3LYP-D3­(BJ)/def2-SVP level.

c Assignments were studied by the IFCT (Hirshfeld) method at the optimized S_0_ structures in toluene.

d The net MLCT is defined as MLCT – LMCT.

The spin–orbit coupling (SOC)-TD-DFT method was then applied to estimate the τ_rad_ and *k*_r_ values of all the studied Pt­(II) complexes based on their optimized S_0_ and T_1_ structures.^56–59^ For conciseness, only the arithmetic-averaged results are discussed herein. The τ_rad_ values for the T_1_ → S_0_ emission of complexes **Pt2**, **Pt3**, **Pt3Ag**, **Pt4**, and **Pt5** were calculated to be 2.10, 2.53, 1.93, 2.66, and 5.37 μs (at the S_0_ structures) (Table 4), respectively, which agree well with the experimental values of 2.63, 3.18, 3.67, 3.00, and 4.65 μs (Table 2), with an MAD of 0.80 μs. In contrast, the calculated τ_rad_ values (4.34, 4.66, 8.16, 10.3, and 8.75 μs) at the T_1_ structures deviate poorly from the experimental values with a larger MAD of 3.81 μs. This suggests that the emission of the studied complexes originates more likely near the S_0_ structure rather than the T_1_ structure, to which the NTO pairs for S_0_ → T_1_ excitations based on the T_1_ structures are depicted in Figure S6.

To obtain a more detailed understanding of the Ag···Pt interactions in **Pt3Ag**, the Hirshfeld method^60^ was used to analyze the contributions of the Ag­(I) metal ion to the selected NTO pairs (at the optimized S_0_ structure, Figure 7). The Ag­(I) metal ion contributed similarly to the virtual NTO (3.2%) and occupied NTO (3.7%). Furthermore, analysis using the IFCT method revealed that the charge transfer from Ag to Pt was 0.5%, while the reverse transfer from Pt to Ag amounted to 0.7%. These negligible CT and NTO contributions suggest no significant electronic interaction between Ag­(I) and Pt­(II) metal ions, which aligns well with the experimental observations.

**Table 4:** Predicted Adiabatic Emission Energy of the T_1_ → S_0_ Transition, Radiative Lifetime (τ_rad_), and Radiative Rate (*k*_r_) Calculated for the Studied Complexes in Toluene^a^ ^,^ ^b^

	λ [nm/eV]	τ_rad_ (μs)	*k*_r_ (10^5^ s^–1^)
**Pt2**	465/2.66	2.10/1.80 (***4.34*/*4.07***)	4.77/5.55 (***2.31*/*2.46***)
**Pt3**	478/2.59	2.53/2.57 (***4.66*/*4.72***)	3.96/3.88 (***2.14*/*2.12***)
**Pt3Ag**	469/2.64	1.93/3.34 (***8.16*/*8.60***)	5.17/3.00 (***1.23*/*1.16***)
**Pt4**	496/2.50	2.66/2.65 (***10.3*/*10.3***)	3.76/3.77 (***0.97*/*0.97***)
**Pt5**	446/2.78	5.37/8.53 (***8.75*/*8.59***)	1.86/1.17 (***1.14*/*1.16***)

a Adiabatic emission energies were calculated by using the optimized T_1_ and S_0_ structures in toluene at the B3LYP-D3­(BJ)/def2-SVP level.

b Computed τ_rad_ and *k*_r_ (arithmetic/Boltzmann averages at 298 K) based on SOC substates at optimized S_0_ (normal font) and T_1_ (italic and bold font in parentheses) structures in toluene.

### Device Fabrication and Characterization

To assess the electroluminescence (EL) of these Pt­(II) complexes, we selected **Pt2** and **Pt5** as the representative emitters for the fabrication of organic light-emitting diodes (OLEDs) employing the vacuum deposition method. As shown in Figure 8a, these OLED devices utilize a multilayered architecture of ITO/HAF-CN (10 nm)/BCFN (60 nm)/SiCzCz: SiTrzCz2 (2:1): emitter (x wt %) (35 nm)/mSiTrz (5 nm)/mSiTrz: Liq (50 wt %) (30 nm)/Liq (1 nm)/Al (100 nm), where ITO, HAT-CN (2,3,6,7,10,11-hexacyano-1,4,5,8,9,12-hexaazatriphenylene), and BCFN (*N*-([1,1′-biphenyl]-4-yl)-9,9-dimethyl-*N*-(4-(9-phenyl-9*H*-carbazol-3-yl)­phenyl)-9*H*-fluoren-2-amine) serve as the anode, hole injection layer, and hole transport layer, respectively. A mixed host consisting of *p*-type SiCzCz (9-(3-(triphenylsilyl)­phenyl)-9*H*-3,9’-bicarbazole) and *n*-type SiTrzCz2 (9,9’-(6-(3-(triphenylsilyl)­phenyl)-1,3,5-triazine-2,4-diyl)­bis­(9H-carbazole)) was employed as the emissive layer (EML) to broaden the recombination zone and facilitate bipolar charge transport capability.^12^ A buffer layer of mSiTrz (2-phenyl-4,6-bis­(3-(triphenylsilyl)­phenyl)-1,3,5-triazine) was inserted between EML and Liq (8-quinolinolato lithium), which served as both the electron transporting layer (ETL) and hole blocking layer,^15^ to confine holes within the EML due to its deepened HOMO level.^61^ The molecular structures of the employed organic materials are depicted in Figure S7 of Supporting Information.

**Figure 8:**
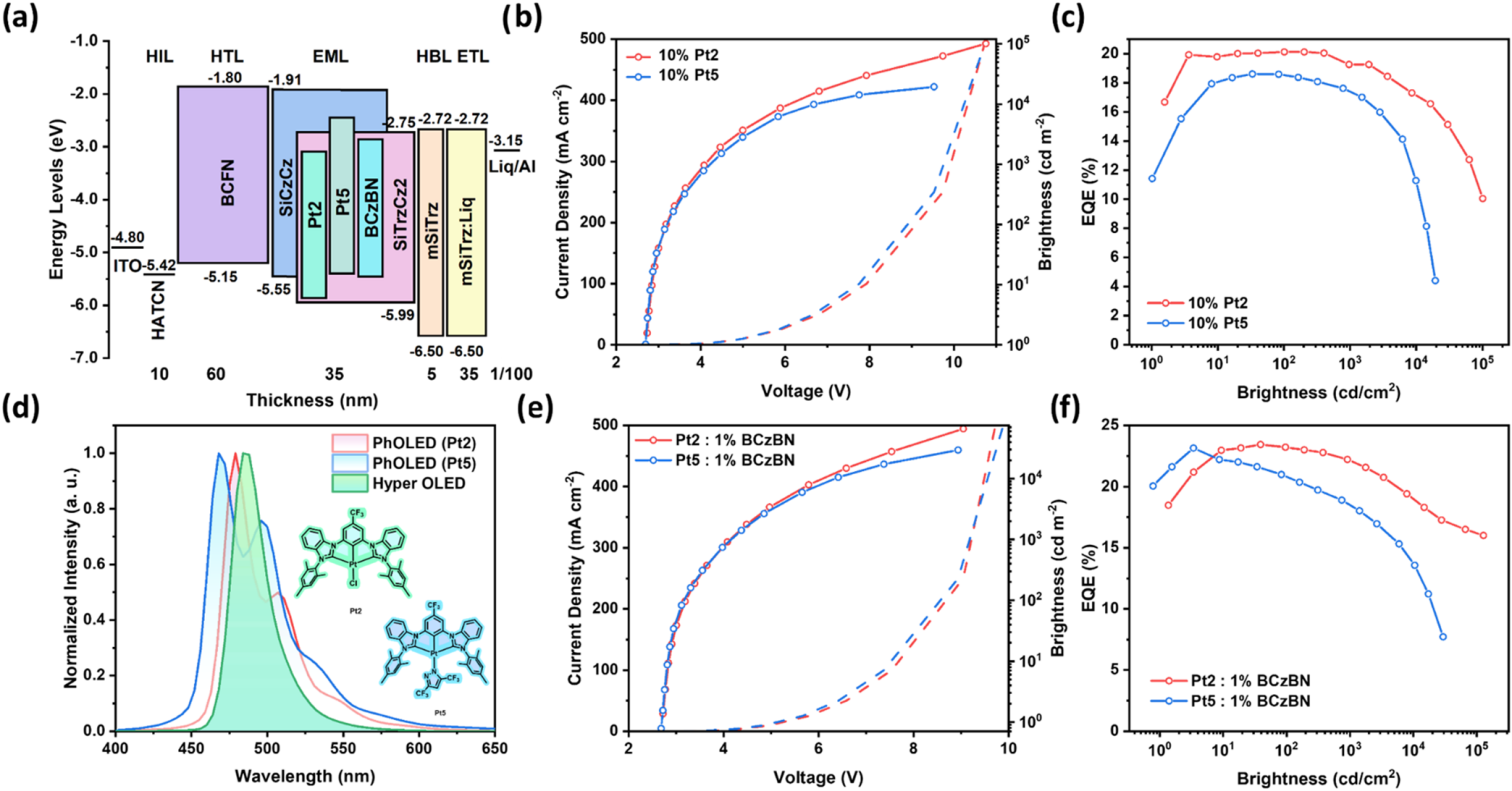
(a) Schematic diagram of the device structure and energy levels (in eV). (b) Current density–voltage–luminance (J–V–L) curves of the PhOLEDs. (c) External quantum efficiency of PhOLEDs. (d) Electroluminescence spectra of PhOLED and hyper-OLED devices (inset: structures of phosphors **Pt2** and **Pt5**). (e) Current density–voltage–luminance (J–V–L) curves of the respective hyper-OLED devices. (f) External quantum efficiency of PhOLEDs.

The doping ratios of **Pt2** and **Pt5** (x wt %) were systematically varied from 5 to 20 wt % in the EML during our initial evaluation (Figure S8). Their EL spectral patterns are in line with their PL profiles in solution, with a slight red shift attributed to the differences in media polarity. In the case of **Pt5**, the emergence of a minor EL shoulder at ∼400 nm may be attributed to exciplex emission from the SiCzCz-SiTrzCz2 cohost.^16^ This observation suggests the incomplete energy transfer from the cohost to the **Pt5** emitter. In sharp contrast to other Pt­(II) emitters featuring alkyl-substituted carbene pincer chelates,^32,33^ **Pt2** and **Pt5** devices retain a well-defined monomer emission at a high doping ratio of 20 wt %, demonstrating effective suppression of excimer emission. This observation further highlights the important role of the N-mesityl substituents of pincer chelate in inhibiting intermolecular Pt···Pt interactions, thereby underscoring the success of our design strategy.

Moreover, **Pt2-**based devices exhibited an outstanding external quantum efficiency (EQ*E*_max_) of 20.1% and a current efficiency (C*E*_max_) of 40.5 cd A^–1^ at a doping ratio of 10 wt %. It also exhibited an ultrahigh maximum luminance (*L*_max_) of 101,000 cd m^–2^ (Figure 8b, Table 5), consistent with the near-unit photoluminescence quantum yield and very fast radiative transition process. Notably, the EQ*E*_max_ decreases to 18.8% at 5 wt %, suggesting inefficient exciton harvesting by the phosphors. On the other hand, the efficiency also decreased to 17.6% at a higher doping ratio of 20 wt %, indicating increased concentration quenching and exciton annihilation (Table S4).^16^ In stark contrast, devices featuring the **Pt5** emitter exhibited a slightly lower EQ*E*_max_ of 18.6% and a much more severe roll-off (Figure 8c), with *L*_max_ decreasing to 19,420 cd m^–2^. This reduction in brightness compared to that of **Pt2-**based devices is ascribed to both the inferior photophysical parameters (*k*_r_, PLQY) and unfavorable charge-injection and transport dynamics. **Pt5** possesses a relatively shallow HOMO (−5.36 eV), which promotes strong hole trapping, while its comparatively high LUMO (−2.33 eV) creates a significant barrier to electron injection. The resulting charge imbalance and excess hole accumulation, combined with inefficient electron capture, enhance polaron formation and lead to pronounced triplet-polaron annihilation (TPA),^62^ thereby limiting device performance.

**Table 5:** Phosphorescent and Hyper-OLED Device Performance Based on **Pt2** and **Pt5** Phosphors and a **BCzBN** Terminal Emitter

emitter	λ_max_ (nm)^a^	fwhm	*V*_on_ (V)^b^	CIE (x,y)^c^	*L*_max_ (cd·m^–2^)	CE (cd·A^–1^)^d^	PE (lm·W^–1^)^d^	EQE (%)^d^
**10 wt % Pt2**	480, 508, 543	27	2.7	0.127, 0.372	101,000	40.5/38.6/32.7	43.2/29.7/15.1	20.1/19.3/16.5
**10 wt % Pt5**	468, 496, 530	49	2.7	0.140, 0.275	19,420	33.4/30.2/19.8	36.3/21.1/9.3	18.6/17.0/11.3
**10 wt % Pt2 /1 wt % BCzBN**	484	27	2.7	0.086, 0.340	127,600	38.8/35.3/29.6	41.4/24.8/14.2	23.4/21.6/18.3
**10 wt % Pt5 /1 wt % BCzBN**	484	29	2.7	0.094, 0.304	29,450	34.8/28.3/21.0	38.9/20.3/10.3	23.2/18.0/13.6

a EL peak max. was recorded at a luminance of 1000 cd·m^–2^, and fwhm indicates the full width at half maxima (in nm).

b Turn-on voltage at 1 cd·m^–2^.

c CIE coordinates at a luminance of 1000 cd·m^–2^.

d Maximum value and values recorded at 1000 cd·m^–2^ and 10000 cd·m^–2^.

To further improve the device performance and color purity, a hyper-OLED architecture was implemented^63,64^ by introducing **BCzBN** as an MR-TADF terminal emitter for both Pt­(II) phosphors. It is noteworthy that the EL spectrum of the fabricated hyper-OLED device exhibits a distinct hypsochromic shift (484 nm vs 490 nm) and a narrowed fwhm (27 nm vs 32 nm) (Figure 8d) compared to the reported **BCzBN-**based TADF OLEDs.^65^ This phenomenon can be related to the fundamentals of the phosphor-sensitized fluorescence (PSF) process, in which the terminal emitter is not the primary site of exciton recombination.^66–68^ It avoids the direct formation of charge-transfer states on the MR-TADF molecule, which is highly sensitive to host polarity, thereby causing bathochromic shifts and spectral broadening. Consequently, the hyper-OLED reveals the unperturbed emission of the MR-TADF core. This also implies efficient Förster resonance energy transfer (FRET) to the terminal emitter and a well-optimized exciton recombination zone,^69,70^ further highlighting the superiority of our molecular design. The resulting hyper-OLED device featuring 10 wt % **Pt2** sensitizer and 1 wt % of **BCzBN** terminal emitter achieved a narrowband emission at 484 nm, and with satisfactory EQ*E*_max_ of 23.4% and *L*_max_ of 127,600 cd m^–2^ (Figure 8e). Notably, the hyper-OLED device featuring **Pt5** sensitizer displays a comparable EQ*E*_max_ of 23.2% (Figure 8f) and a notably reduced *L*_max_ of 29,450 cd m^–2^, as the relatively long radiative lifetime of **Pt5** enables more efficient FRET to the terminal emitter and compensates the initially lowered EQE of PhOLED devices.^69^

## Conclusion

In summary, we have developed an effective strategy for synthesizing blue Pt­(II) phosphors with suppressed excimer emission by incorporating N-mesityl appendages into their dicarbene pincer chelate. Functionalization of these Pt­(II) complexes is possible, and the chloride ligand of the parent Pt­(II) complexes **Pt1** and **Pt2** could be easily replaced with pyrazolate to give Pt­(II) derivatives **Pt3**–**Pt5**, as well as the “AgCl” adducts **Pt3Ag** and **Pt4Ag**. These Pt­(II) complexes exhibit superior photophysical properties and are suitable for the fabrication of sky-blue PhOLED devices with excellent performance. Alternatively, the coordination of Ag­(I) (or AgCl) to Pt­(II) metal ions had minimal influence on the photophysical behaviors, which aligned well with both the experimental and theoretical results. As a representative example, a PhOLED device based on **Pt2** delivered phosphorescence with a max. EQE of 20.1% and max. luminance reaching ∼100,000 cd·m^–2^. Moreover, integrating **Pt2** into a phosphor-sensitized fluorescence (or hyperphosphorescence) system facilitates efficient energy transfer to the MR-TADF terminal emitter **BCzBN**. The resulting hyper-OLED device successfully harmonizes high brightness and efficiency with excellent color chromaticity, showcasing outstanding roll-off resistance at higher driving current densities (EQE of 21.6% at 1000 cd m^–2^ and 18.3% at 10,000 cd·m^–2^). These findings establish a general design principle for Pt­(II) dicarbene pincer complexes and highlight their potential application for next-generation high-performance blue OLEDs.

## Experimental Section

### General Information and Materials

Commercially available reagents were used without further purification. All solvents were dried and degassed before use, and all reactions were conducted under an argon atmosphere and monitored using precoated TLC plates (0.20 mm with fluorescent indicator F254). No unusual hazards were noted. All NMR spectra (^1^H, ^19^F, ^195^Pt) were recorded with a Bruker 400 MHz “AVANCE NEO” instrument, and the ^195^Pt NMR spectral data were referenced to an external standard of a 1 M solution of Na_2_PtCl_6_ in D_2_O. High-resolution mass spectra were obtained using a Bruker microTOF-Q instrument with acetonitrile as the solvent. TGA measurements were performed on a TA Instrument TGAQ50 at a heating rate of 10 °C min^–1^ under a nitrogen atmosphere. Single-crystal X-ray structural analyses were conducted using phi and omega scan modes (APEX3) on a Bruker D8 Venture Photon II diffractometer with microfocus X-ray sources at 233 K.

#### Synthesis of Pt1

To a 100 mL reaction flask were added **C1** (0.72 g, 0.81 mmol), K_2_PtCl_4_ (0.30 g, 0.72 mmol), K_2_CO_3_ (1.0 g, 7.2 mmol), and propionic acid (60 mL). The mixture was heated to reflux overnight. Then, the solvent was removed under reduced pressure, and the residue was dissolved in 50 mL of CH_2_Cl_2_, neutralized with a saturated solution of NaHCO_3_, washed with deionized water, and dried over anhydrous Na_2_SO_4_. The crude product was further purified via silica gel column chromatography using a mixture of hexane/ethyl acetate/CH_2_Cl_2_ (6/1/1, v/v/v) as the eluent to afford a yellow solid of **Pt1**. Yield: 340 mg, 56%.

Selected spectroscopic data of **Pt1**: HRMS (ESI) for C_42_H_41_PtN_4_Cl [M + CH_3_CN–Cl]^+^: calcd 837.3239, found 837.3236; ^1^H NMR (400 MHz, CDCl_3_) δ 8.11 (d, *J* = 8.0 Hz, 2H), 7.66 (t, *J* = 7.6 Hz, 2H), 7.53 (t, *J* = 8.0 Hz, 2H), 7.31 (t, *J* = 8.0 Hz, 2H), 6.99 (s, 4H), 6.94 (d, *J* = 8.0 Hz, 2H), 2.32 (s, 6H), 1.97 (s, 12H), 1.62 (s, 9H). ^195^Pt NMR (86 MHz, CDCl_3_) δ−3983 (s, 1Pt).

#### Synthesis of Pt2

The procedure was analogous to that described for **Pt1**. **Pt2** was obtained from **C2** (0.46 g, 0.51 mmol) and K_2_PtCl_4_ (0.20 g, 0.48 mmol) as a yellow solid. Yield: 236 mg, 58%.

Selected spectroscopic data of **Pt2**: HRMS (ESI) for C_39_H_32_PtN_4_F_3_Cl [M + CH_3_CN–Cl]^+^: calcd 849.2487, found 849.2502; ^1^H NMR (400 MHz, CDCl_3_) δ 8.13 (d, *J* = 8.0 Hz, 2H), 7.82 (t, *J* = 8.0 Hz, 2H), 7.58 (t, *J* = 8.0 Hz, 2H), 7.36 (t, *J* = 8.0 Hz, 2H), 7.00 (s, 4H), 6.98 (d, *J* = 8.0 Hz, 2H), 2.33 (s, 6H), 1.97 (s, 12H). ^19^F NMR (376 MHz, CDCl_3_) δ−60.88 (s, 3F). ^195^Pt NMR (86 MHz, CDCl_3_) δ−3924 (s, 1Pt).

Selected crystal data of **Pt2**: CCDC number: 2517642. C_39_H_32_PtN_4_F_3_Cl; M = 844.22; tetragonal; space group 88: I4/*a*; *a* = 36.6211(8) Å, *b* = 36.6211(8) Å, *c* = 10.2108(3) Å; *V* = 13693.8(7) Å^3^; *Z* = 16; ρ_Calcd_ = 1.638 g·cm^–3^; μ = 8.804 mm^–1^; F(000) = 6656, λ­(Cu–K_α_) = 1.54178 Å; T = 193(2) K; crystal size/mm^3^: 0.01 × 0.02 × 0.28; 98701 reflections collected, 7008 independent reflections (*R*_int_ = 0.0656), data/restraints/parameters = 7008/182/475, GOF = 1.030, final R_1_[*I* > 2σ­(*I*)] = 0.0335 and *w*R_2_(all data) = 0.0886.

#### Synthesis of Pt3

To a 50 mL reaction flask, **Pt1** (120 mg, 0.144 mmol), 3,5-diphenylpyrazole (35 mg, 0.16 mmol), Na_2_CO_3_ (34 mg, 0.32 mmol), and ethanol (20 mL) were added. The mixture was then heated to reflux overnight. Upon completion, the mixture was cooled to RT, and the resulting yellow precipitate was collected. This precipitate was then dissolved in 50 mL of CH_2_Cl_2_, washed with deionized water, filtered through Celite, and concentrated to dryness under vacuum. The crude product was further recrystallized from a mixture of CH_2_Cl_2_ and methanol to yield yellow crystals of **Pt3**. Yield: 128 mg, 87%.

Selected spectroscopic data of **Pt3**: HRMS (ESI) for C_57_H_52_PtN_6_ [M + 1]^+^: calcd 1016.3903, found 1016.3981; ^1^H NMR (400 MHz, CDCl_3_) δ 8.12 (d, *J* = 8.0 Hz, 2H), 7.88 (d, *J* = 7.6 Hz, 2H), 7.81 (d, *J* = 7.2 Hz, 2H), 7.70 (t, *J* = 5.6 Hz, 2H), 7.49 (t, *J* = 7.6 Hz, 2H), 7.34 (t, *J* = 7.6 Hz, 2H), 7.24 (t, *J* = 7.6 Hz, 2H), 7.15 (t, *J* = 7.2 Hz, 1H), 7.02 (t, *J* = 8.0 Hz, 2H), 6.90 (t, *J* = 7.2 Hz, 1H), 6.85 (d, *J* = 8.0 Hz, 2H), 6.27 (s, 2H), 6.18 (s, 1H), 6.16 (s, 2H), 2.07 (s, 6H), 1.89 (s, 6H), 1.63 (s, 9H), 1.33 (s, 6H). ^195^Pt NMR (86 MHz, CDCl_3_) δ−4053.34 (s, 1Pt). ^195^Pt NMR (86 MHz, CDCl_3_) δ−4057 (s, 1Pt).

Selected crystal data of **Pt3**: CCDC number: 2517643. C_57_H_52_N_6_Pt; M = 1016.13; monoclinic; space group 15: *C2*/c; *a* = 19.2338(10) Å, *b* = 15.7406(7) Å, *c* = 17.2715(9) Å; β = 116.548(2)°; *V* = 4677.6(4) Å^3^; *Z* = 4; ρ_Calcd_ = 1.443 g·cm^–3^; μ = 5.951 mm^–1^; F(000) = 2056, λ­(Cu-K_α_) = 1.54178 Å; T = 213(2) K; crystal size/mm^3^: 0.03 × 0.12 × 0.26; 82949 reflections collected, 4741 independent reflections (*R*_int_ = 0.0507), data/restraints/parameters = 4741/420/421, GOF = 1.103, final R_1_[*I* > 2σ­(*I*)] = 0.0312 and *w*R_2_(all data) = 0.0771.

#### Synthesis of Pt3Ag

To a 50 mL reaction flask, **Pt1** (50 mg, 0.059 mmol), 3,5-diphenylpyrazole (14 mg, 0.065 mmol), Ag_2_O (27 mg, 0.12 mmol), and CH_2_Cl_2_ (10 mL) were added. The mixture was then heated to reflux overnight. Then, it was filtered through Celite, and the filtrate was diluted with CH_2_Cl_2_, washed with deionized water, and concentrated to dryness under vacuum. The crude product was further recrystallized from a mixture of CH_2_Cl_2_ and methanol to yield yellow crystals of **Pt3Ag**. Yield: 58 mg, 85%.

Selected spectroscopic data of **Pt3Ag**: HRMS (ESI) for C_57_H_52_PtN_6_AgCl [M + 1]^+^: calcd 1159.2637, found 1159.4758; ^1^H NMR (400 MHz, CDCl_3_) δ 8.16 (d, *J* = 8.4 Hz, 2H), 7.98 (br, 2H), 7.80 (dd, *J* = 8.4 Hz, 1.2 Hz, 2H), 7.72 (t, *J* = 6.0 Hz, 2H), 7.54 (dt, *J* = 8.4 Hz, 1.2 Hz, 2H), 7.44 (tt, *J* = 8.0 Hz, 2.0 Hz, 2H), 7.33–7.23 (m, 3H), 7.12 (t, *J* = 7.6 Hz, 2H), 7.06 (tt, *J* = 7.2 Hz, 1.6 Hz, 1H), 6.82 (d, *J* = 8.0 Hz, 2H), 6.40 (s, 2H), 6.31 (d, *J* = 3.2 Hz, 1H), 6.17 (s, 2H), 2.05 (s, 6H), 2.04 (s, 6H), 1.68 (s, 9H), 1.26 (s, 6H). ^195^Pt NMR (86 MHz, CDCl_3_) δ−4131 (s, 1Pt).

Selected crystal data of **Pt3Ag**: CCDC number: 2517644. C_57_H_52_AgClN_6_Pt; M = 1159.45; triclinic; space group 2: *P*-1; *a* = 11.2500(4) Å, *b* = 11.6449(4) Å, *c* = 18.7122(6) Å; α = 96.1300(10)°; β = 92.9200(10)°; γ = 92.6350(10)°; *V* = 2430.95(14) Å^3^; *Z* = 2; ρ_Calcd_ = 1.584 g·cm^–3^; μ = 3.377 mm^–1^; F(000) = 1156, λ­(Mo–K_α_) = 0.71073 Å; T = 193(2) K; crystal size/mm^3^: 0.03 × 0.18 × 0.34; 60022 reflections collected, 9976 independent reflections (*R*_int_ = 0.076), data/restraints/parameters = 9976/0/604, GOF = 1.051, final R_1_[*I* > 2σ­(*I*)] = 0.0314 and *w*R_2_(all data) = 0.0779.

#### Synthesis of Pt4

The procedure was analogous to that described for **Pt3**. The respective **Pt4** was obtained from **Pt2** (120 mg, 0.144 mmol) and 3,5-diphenylpyrazole (35 mg, 0.159 mmol) as yellow solids. Yield: 116 mg, 78%. This Pt­(II) complex was purified by recrystallization from a mixed CH_2_Cl_2_ and methanol solution and vacuum sublimation.

Selected spectroscopic data of **Pt4**: HRMS (ESI) for C_54_H_43_PtN_6_F_3_ [M + 1]^+^: calcd 1028.3151, found 1028.3234; ^1^H NMR (400 MHz, CDCl_3_) δ 8.14 (d, *J* = 8.0 Hz, 2H), 7.85 (t, *J* = 6.0 Hz, 1H), 7.81 (t, *J* = 7.2 Hz, 4H), 7.54 (t, *J* = 8.0 Hz, 2H), 7.35 (t, *J* = 7.6 Hz, 2H), 7.29 (t, *J* = 8.0 Hz, 2H), 7.17 (t, *J* = 7.2 Hz, 1H), 7.03 (t, *J* = 7.6 Hz, 2H), 6.92 (t, *J* = 7.2 Hz, 1H), 6.88 (d, *J* = 8.0 Hz, 2H), 6.28 (s, 2H), 6.18 (s, 1H), 6.17 (s, 2H), 2.08 (s, 6H), 1.89 (s, 6H), 1.29 (s, 6H). ^19^F NMR (376 MHz, CDCl_3_) δ−60.80 (s, 3F). ^195^Pt NMR (86 MHz, CDCl_3_) δ− 4011 (s, 1Pt).

Selected crystal data of **Pt4**: CCDC number: 2517645. C_55.50_H_48_ClF_3_N_6_OPt; M = 1102.54; monoclinic; space group 14: *P2*_1_ */n*; *a* = 14.6205(5) Å, *b* = 18.0748(6) Å, *c* = 18.4143(6) Å; β = 91.3760(10)°; *V* = 4864.8(3) Å^3^; *Z* = 4; ρ_Calcd_ = 1.505 g·cm^–3^; μ = 6.372 mm^–1^; F(000) = 2212, λ­(Cu-K_α_) = 1.54178 Å; T = 193(2) K; crystal size/mm^3^: 0.04 × 0.17 × 0.22; 84694 reflections collected, 9957 independent reflections (*R*_int_ = 0.0482), data/restraints/parameters = 9957/72/659, GOF = 1.035, final R_1_[*I* > 2σ­(*I*)] = 0.0250 and *w*R_2_(all data) = 0.0703.

#### Synthesis of Pt4Ag

The procedure was analogous to that described for **Pt3Ag**. The respective **Pt4Ag** was obtained from **Pt2** (50 mg, 0.059 mmol) and 3,5-diphenylpyrazole (14 mg, 0.065 mmol) as a yellow solid. Yield: 53 mg, 76%.

Selected spectroscopic data of **Pt4Ag**: ^1^H NMR (400 MHz, CDCl_3_) δ 8.18 (d, *J* = 8.4 Hz, 2H), 7.93 (br, 2H), 7.88 (t, *J* = 6.8 Hz, 2H), 7.78 (d, *J* = 8.4 Hz, 2H), 7.58 (t, *J* = 8.0 Hz, 2H), 7.44 (t, *J* = 7.6 Hz, 2H), 7.36–7.28 (m, 3H), 7.14 (t, *J* = 7.6 Hz, 2H), 7.08 (t, *J* = 7.2 Hz, 1H), 6.86 (d, *J* = 8.0 Hz, 2H), 6.42 (s, 2H), 6.33 (d, J = 2.8 Hz, 1H), 6.18 (s, 2H), 2.05 (s, 6H), 2.03 (s, 6H), 1.29 (s, 6H). ^19^F NMR (376 MHz, CDCl_3_ CDCl_3_) δ−61.09 (s, 3F). ^195^Pt NMR (86 MHz, CDCl_3_) δ−4089 (s, 1Pt).

#### Synthesis of Pt5

The procedure was analogous to that described for **Pt3**. The respective **Pt5** was obtained from **Pt2** (50 mg, 0.059 mmol) and 3,5-bis­(trifluoromethyl)­pyrazole (13 mg, 0.065 mmol) as yellow solids. Yield: 54 mg, 90%.

Selected spectroscopic data of **Pt5**: HRMS (ESI) for C_54_H_43_PtN_6_F_3_ [M + 1]^+^: calcd 1013.2306, found 1013.2396; ^1^H NMR (400 MHz, CDCl_3_) δ 8.13 (d, *J* = 8.4 Hz, 2H), 7.81 (t, *J* = 6.4 Hz, 2H), 7.56 (t, *J* = 8.4 Hz, 2H), 7.34 (t, *J* = 8.0 Hz, 2H), 6.90 (d, *J* = 8.0 Hz, 2H), 6.70 (s, 2H), 6.59 (s, 2H), 5.89 (s, 1H), 2.17 (s, 6H), 1.94 (s, 6H), 1.78 (s, 6H). ^19^F NMR (376 MHz, CDCl_3_) δ−57.36 (t, *J* = 5.6 Hz, 3F), −60.48 (s, 3F), −61.05 (s, 3F). ^195^Pt NMR (86 MHz, CDCl_3_) δ−4104 (s, 1Pt).

Selected crystal data of **Pt5**: CCDC number: 2517646. C_44_H_33_F_9_N_6_Pt; M = 1011.85; monoclinic; space group 14: *P2*_1_ */n*; *a* = 11.5405(4) Å, *b* = 14.1824(5) Å, *c* = 24.2259(8) Å; β = 95.8090(10)°; *V* = 3944.7(2) Å^3^; *Z* = 4; ρ_Calcd_ = 1.704 g·cm^–3^; μ = 3.641 mm^–1^; F(000) = 1992, λ­(Mo-K_α_) = 0.71073 Å; T = 173(2) K; crystal size/mm^3^: 0.03 × 0.19 × 0.41; 48441 reflections collected, 8128 independent reflections (*R*_int_ = 0.0584), data/restraints/parameters = 8128/72/574, GOF = 1.013, final R_1_[*I* > 2σ­(*I*)] = 0.0297 and *w*R_2_(all data) = 0.0716.

#### Conversion from Pt3 to Pt3Ag

To a 50 mL flask, **Pt3** (25 mg, 0.025 mmol), AgPF_6_ (13 mg, 0.051 mmol), and CH_2_Cl_2_ (6 mL) were added. The mixture was heated at reflux for 2 h. Then, the mixture was cooled to RT, ^*n*^Bu_4_NCl (34 mg, 0.12 mmol) was added, and refluxed for another 1 h. After cooling to RT, the mixture was filtered through Celite, washed with deionized water, and concentrated to dryness under vacuum. The crude product was further recrystallized from a mixture of CH_2_Cl_2_ and methanol to yield yellow crystals of **Pt3Ag**. The conversion was confirmed using NMR spectroscopic methods. Yield: 22 mg, 77%.

## Supplementary Material



## Notes

Synthetic procedures and spectroscopic data of chelates and associated intermediates; TD-DFT investigations; selected analytical data; and nonessential OLED data of studied Pt­(II) emitters (PDF)
