# Supplementary material for: Platinum(II) Complexes with Carbene Pincer Chelates for Blue Hyperphosphorescent Organic Light-Emitting Diodes
Source: Inorg Chem. 2026 Jun 22;65(26):15114–25. doi: 10.1021/acs.inorgchem.6c01873 (PMC13343508; doi:10.1021/acs.inorgchem.6c01873)
Supplement: Supplementary file 1 [file ic6c01873_si_001.pdf]

Supporting Information

Platinum(II) complexes with carbene pincer chelate for blue hyperphosphorescent organic light-emitting diodes

Guowei Ni,[a,] Yufeng Sang,[a,] Lin Cheng,[b,] Wei He,[c,] Shek-Man Yiu,[b] Kai Chung Lau,[b,*] Guodan Wei,[c,*] Yun Chi,[a,b,*]

 G. Ni, Y. Sang, L. Cheng and W. He contributed equally to this work.

(a) Department of Materials Science and Engineering, and Center of Super-Diamond and Advanced Films (COSDAF), City University of Hong Kong, Hong Kong SAR, E-mail: yunchi@cityu.edu.hk.

(b) Department of Chemistry, City University of Hong Kong, Hong Kong SAR, E-mail: kaichung@cityu.edu.hk.

(c) Institute of Materials Research, Tsinghua Shenzhen International Graduate School, Tsinghua University, Shenzhen 518055, China, E-mail: weiguodan@sz.tsinghua.edu.cn

Experimental section:

General information and materials. Commercially available reagents were used without further purification. All solvents were dried and degassed before used, and all reactions were conducted under argon atmosphere and monitored using pre-coated TLC plates (0.20 mm with fluorescent indicator F254). All NMR spectra (^{1}H, ^{19}F, ^{195}Pt) were recorded with Bruker 400 MHz "AVANCE NEO" instrument and ^{195}Pt NMR spectral data were referenced to an external standard of 1M solution of Na_2PtCl_6 in D_2O. The high-

resolution mass spectra were obtained using Bruker microTOF-Q instrument and acetonitrile as solvent. TGA measurements were performed on a TA Instrument TGAQ50, at a heating rate of 10 °C min^{-1} under nitrogen atmosphere. The single crystal X-ray structural analyses were conducted using phi and omega scans mode (APEX3) on a Bruker D8 Venture Photon II diffractometer with microfocus X-ray sources at 233 K.

Photophysical measurements: All photophysical measurements in this study were performed at room temperature (298 K). UV-visible spectra were recorded on HITACHI UH4150 instrument. The emission spectra of the solution state were measured with an Edinburgh FLS 1000 instrument. Both wavelength-dependent excitation and emission responses were calibrated. Steady-state absorption and emission spectra of the studied complexes were measured in toluene at RT, where spectroscopic grade solvents were employed. To specify the quantum yield in the fluid state, samples were degassed using at least three freeze-pump-thaw cycles. The solution quantum yields are calculated using coumarin 102 that has a known quantum yield, according to the following equation:

$$\Phi = \Phi_R \frac{I}{I_R} \frac{A_R}{A} \frac{\eta^2}{\eta_R^2}$$

Where Φ is the PL quantum yield, the subscript R refers to the reference compound of known quantum yield, *I* is the integrated fluorescence intensity, and η is the refractive index of solvent. *A* is the absorbance at the excitation wavelength with the measured absorbance between 0.05 - 0.1.

Electrochemistry: Cyclic voltammetry was conducted on a CHI660 Electrochemical Analyzer. All anodic and cathodic peak potentials were referenced to the ferrocene redox couple (Fc/Fc^+ = 0.38 V). Oxidation bands were measured using platinum working electrode with 0.1 M of NBu_4PF_6 as electrolyte in dichloromethane. The potentials were referenced externally to a ferrocene/ferrocenium (Fc/Fc^+) couple.

Computational details of theoretical investigations

The geometries, electronic structures, and electronic excitations of the studied Pt(II) complexes

were investigated at the B3LYP-D3(BJ)/def2-SVP level [1-5] using Gaussian 16 set of programs. [6] The solvent effect of toluene was taken account by the polarizable continuum model (PCM). [7, 8] The structures of the studied Pt(II) complexes were optimized based on their X-ray crystallographic data of **Pt2**, **Pt3**, **Pt3Ag, Pt4** and **Pt5**. Subsequently, the TD–DFT calculations [9, 10] were performed based on their optimized S_0 structures, including the $T_1 \sim T_{100}$ and $S_1 \sim S_{100}$ excited states (200 states in total). For excitations involving multiple orbital contributions (e.g., $S_0 \rightarrow T_1$ excitation in this work), the natural transition orbital (NTO) analysis was employed. [11] The density in the IFCT analysis and the contribution of Pt and Ag metals to the NTO pairs were quantified using the Hirshfeld method in Multiwfn. [12-14]

The spin-orbit coupling (SOC)-TDDFT computations [15] were executed in ORCA (v 6.0.1) software [16, 17] at the optimized S_0 and T_1 structures using the B3LYP functional with ZORA Hamiltonian (SARC-ZORA-TZVP for Pt and Ag; ZORA-def2-SVP for other elements). [18, 19] A total of 200 low-lying excited states (100 for singlet and 100 for triplet) were within the COSMO solvation model for toluene. [20] The radiative lifetime (τ_{rad}) and radiative rate constants (k_r) were derived from the arithmetic average and Boltzmann average (at 298 K) of the SOC substates of the T_1 excited states. [15]

Synthesis of carbene pincer chelates (C1 and C2)

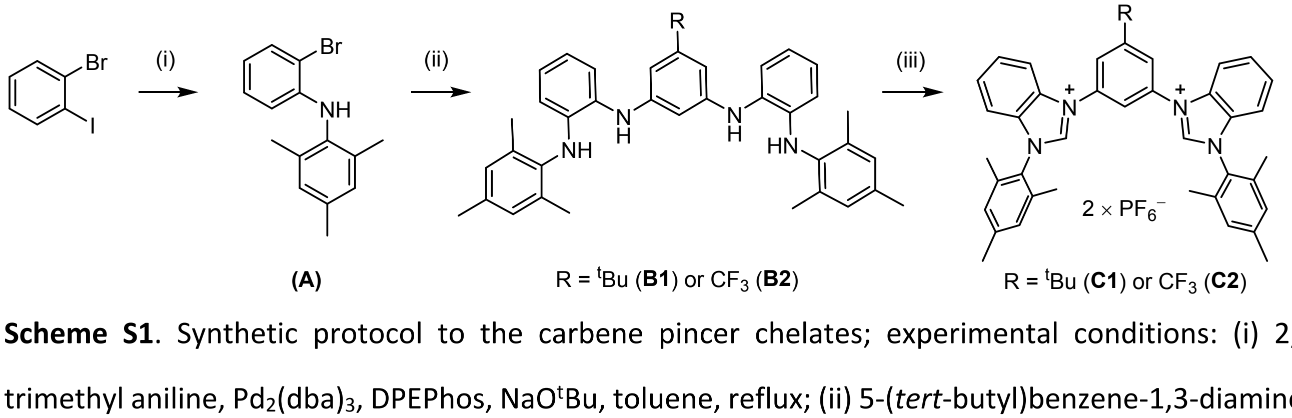

Scheme S1. Synthetic protocol to the carbene pincer chelates; experimental conditions: (i) 2,4,6-trimethyl aniline, $Pd_2(dba)_3$, DPEPhos, NaO^{t}Bu, toluene, reflux; (ii) 5-(*tert*-butyl)benzene-1,3-diamine for **C1** and 5-(trifluoromethyl)benzene-1,3-diamine for **C2**, $Pd_2(dba)_3$, DPEPhos, NaO^{t}Bu, toluene, reflux; (iii) $CH(OEt)_3$, conc. $HCl_{(aq)}$, reflux.

Synthesis of N-(2-bromophenyl)-2,4,6-trimethylaniline (A)

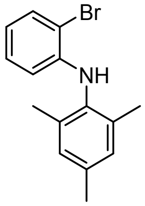

To a 500 mL flask was added 1-bromo-2-iodobenzene (9.2 g, 32.5 mmol), 2,4,6-trimethyl aniline (4.0 g, 29.6 mmol), $Pd_2(dba)_3$ (0.34 g, 0.37 mmol), DPEPhos (1.2 g, 2.2 mmol), NaO^tBu (11.4 g, 0.12 mol) and toluene (240 mL). The mixture was then heated to reflux under argon for 2 hours. After removal of solvent under reduced pressure, the residue was dissolved in ethyl acetate and filtered through Celite®. The filtrate was then washed with deionized water, dried over anhydrous Na_2SO_4, concentrated and further purified by flash silica gel column chromatography eluting with hexane, and then with a mixture of hexane and ethyl acetate (20/1, v/v) in giving a white solid. Yield: 7.51 g, 87%.

Selected spectroscopic data of **A**: ^1H NMR (400 MHz, $CDCl_3$) δ 7.47 (dd, J = 8.0 Hz, 1.6 Hz, 1H), 7.01 (dt, J = 7.6 Hz, 1.6 Hz, 1H), 6.96 (s, 2H), 6.58 (dt, J = 7.6 Hz, 1.6 Hz, 1H), 6.15 (dd, J = 8.0 Hz, 1.6 Hz, 1H), 5.63 (s, 1H), 2.32 (s, 3H), 2.16 (s, 6H).

Synthesis of 5-(tert-butyl)benzene-1,3-diamine

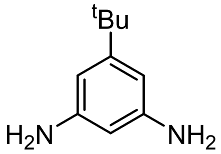

To an 80 mL seal tube was added 25 % NH_4OH (4.6 mL, 67.6 mmol), 1,3-dibromo-5-(tertbutyl) benzene (1.0 g, 3.4 mmol), CuI (0.27 g, 1.4 mmol), L-proline (0.31 g, 2.7 mmol), K_2CO_3 (1.9 g, 13.6 mmol) and DMSO (20 mL). The tube was purged with argon, and then sealed and heated to 130 °C for 24 hours. After cooled to RT, the solution was diluted with 40 mL of ethyl acetate, filtered through Celite® and the filtrate was washed with 200 mL of deionized water. The organic layer was dried over anhydrous Na_2SO_4, concentrated and further purified by silica gel column chromatography, eluting with a mixture of hexane and ethyl acetate (1/1, v/v) in giving a brown solid. Yield: 0.36 g, 64%.

Selected spectroscopic data: ^{1}H NMR (400 MHz, $CDCl_3$) δ 6.17 (d, J = 2.0 Hz, 2H), 5.90 (t, J = 2.0 Hz, 1H), 3.53 (br, 4H), 1.25 (s, 9H).

Synthesis of N^1,N$^{1'}$-(5-(*tert*-butyl)-1,3-phenylene)bis(N^2-mesitylbenzene-1,2-diamine) (B1)

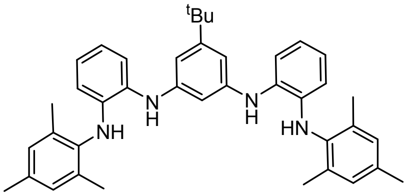

To a 250 mL reaction flask was added **A** (2.0 g, 6.9 mmol), 5-(*tert*-butyl)benzene-1,3-diamine (0.52 g, 3.1 mmol), $Pd_2(dba)_3$ (72 mg, 0.078 mmol), DPEPhos (0.25 g, 0.47 mmol), and NaO^tBu (1.2 g, 12.5 mmol) in anhydrous toluene (100 mL). The reaction mixture was then heated to reflux under argon for 4 hours. After then, the solvent was removed under reduced pressure, and the residue was dissolved in ethyl acetate and filtered through Celite®. The filtrate was then washed with deionized water, dried over anhydrous Na_2SO_4, concentrated and purified by silica gel column chromatography, eluting with a mixture of hexane and ethyl acetate (20/1, v/v) in giving a white solid. Yield: 1.16 g, 64%.

Selected spectroscopic data of **B1**: ^{1}H NMR (400 MHz, $CDCl_3$) δ 7.22 (dd, J = 7.6 Hz, 1.6 Hz, 2H), 6.91 (s, 4H), 6.89 (dt, J = 7.6 Hz, 1.6 Hz, 2H), 6.58 (dt, J = 7.6 Hz, 1.6 Hz, 2H), 6.43 (d, J = 2.0 Hz, 2H), 6.25 – 6.20 (m, 3H), 5.48 (s, 2H), 5.25 (s, 2H), 2.29 (s, 6H), 2.12 (s, 12H), 1.27 (s, 9H).

Synthesis of N^1,N$^{1'}$-(5-(trifluoromethyl)-1,3-phenylene)bis(N^2-mesitylbenzene-1,2-diamine) (B2)

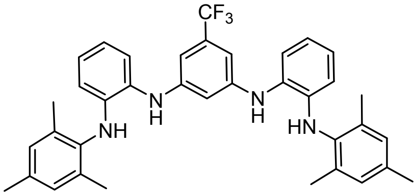

The procedure was analogous to that described for **B1**, and the respective **B2** was obtained from **A** (4.0 g, 13.8 mmol) and 5-(trifluoromethyl)benzene-1,3-diamine (1.1 g, 6.3 mmol) as a white solid. Yield: 2.84 g, 76%.

Selected spectroscopic data of **B2**: ^{1}H NMR (400 MHz, $CDCl_3$) δ 7.16 (d, J = 8.0 Hz, 2H), 6.99 (t, J =

8.0 Hz, 2H), 6.92 (s, 4H), 6.73 (t, *J* = 8.0 Hz, 2H), 6.48 (s, 2H), 6.37 (s, 1H), 6.24 (d, *J* = 8.0 Hz, 2H), 5.45 (s, 2H), 5.40 (s, 2H), 2.30 (s, 6H), 2.12 (s, 12H). ^{19}F NMR (376 MHz, $CDCl_3$) δ -62.98 (s, 3F).

Synthesis of Carbene Pincer Chelate (C1)

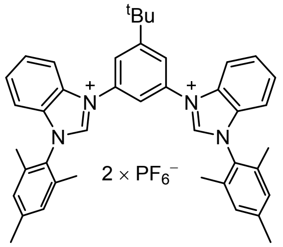

To a 100 mL reaction flask was added **B1** (1.0 g, 1.72 mmol), conc. $HCl_{(aq)}$ (1.5 mL) and triethyl orthoformate (50 mL). The reaction mixture was heated at 90 °C under argon for 4 hours. After then, the solvent was evaporated under reduced pressure, the residue was dissolved in methanol (20 mL), and saturated KPF_6 solution in water was added with vigorous stirring to induce precipitation. The precipitate was filtered and dried under vacuum to obtain an off-white solid of **C1**. Yield: 1.40 g, 91%.

Selected spectroscopic data of **C1**: ^{1}H NMR (400 MHz, DMSO-d_6) δ 10.64 (s, 2H), 8.59 (t, *J* = 2.0 Hz, 1H), 8.38 (d, *J* = 2.0 Hz, 2H), 8.25 (d, *J* = 8.4 Hz, 2H), 7.88 (dt, *J* = 8.4 Hz, 1.2 Hz, 2H), 7.88 (dt, *J* = 8.4 Hz, 1.2 Hz, 2H), 7.60 (d, *J* = 8.4 Hz, 2H), 7.31 (s, 4H), 2.43 (s, 6H), 2.11 (s, 12H), 1.51 (s, 9H). ^{19}F NMR (376 MHz, DMSO-d_6) δ -69.20 (d, *J* = 710 Hz, 12F).

Synthesis of Carbene Pincer Chelate (C2)

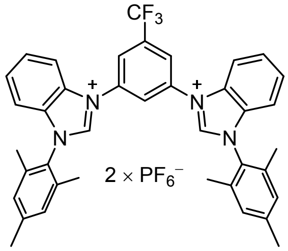

The procedure was analogous to that described for **C1**, and the respective pincer chelate **C2** was obtained from **B2** (1.0 g, 1.68 mmol) as an off-white solid. Yield: 1.4 g, 93%.

Selected spectroscopic data of **C2**: ^{1}H NMR (400 MHz, DMSO-d_6) δ 10.67 (s, 1H), 9.12 (t, *J* = 1.6 Hz,

1H), 8.90 (d, *J* = 1.6 Hz, 2H), 8.30 (d, *J* = 8.4 Hz, 2H), 7.90 (t, *J* = 8.4 Hz, 2H), 7.81 (t, *J* = 8.0 Hz, 2H), 7.61 (d, *J* = 8.0 Hz, 2H), 7.31 (s, 4H), 2.43 (s, 6H), 2.10 (s, 12H). ^{19}F NMR (376 MHz, DMSO-d_6) δ -61.07 (s, 3F), -69.20 (d, *J* = 710 Hz, 12F).

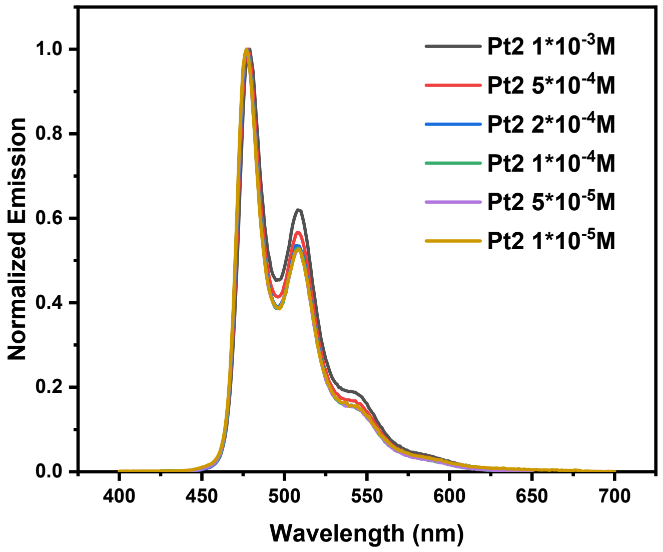

Figure S1. Emission profile of **Pt2** at RT in toluene at various concentrations.

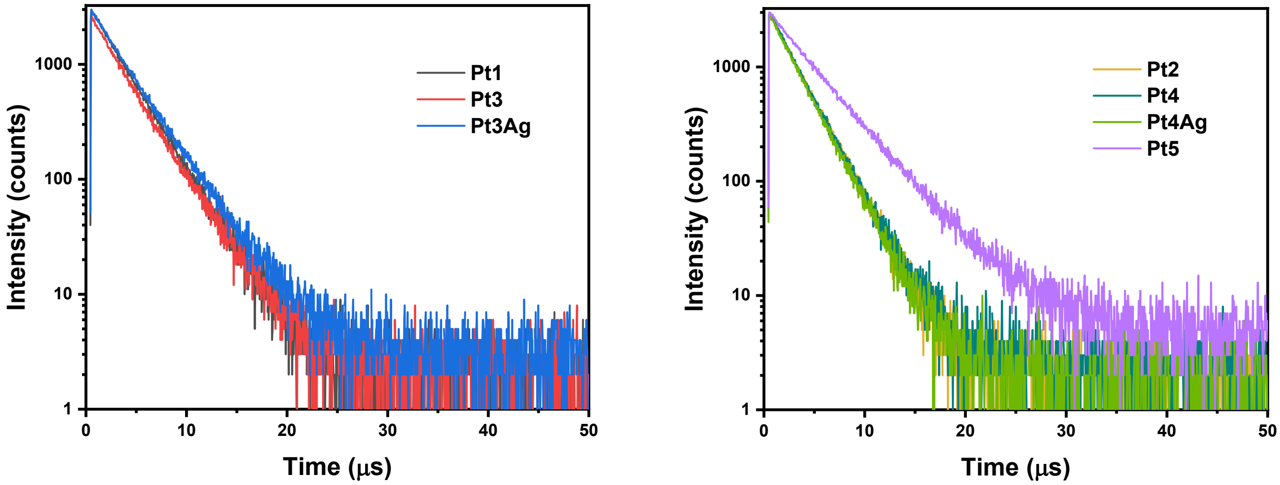

Figure S2. The transient decay profile of studied Pt(II) complexes in degassed toluene at RT (excited at 373 nm).

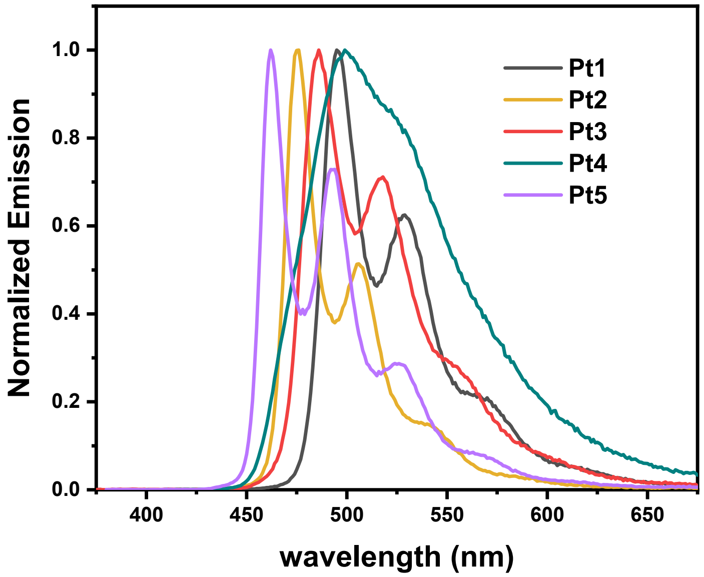

Figure S3. Emission spectra of Pt(II) complexes **Pt1** – **Pt5** in drop-cast PS neat film at 2 wt%.

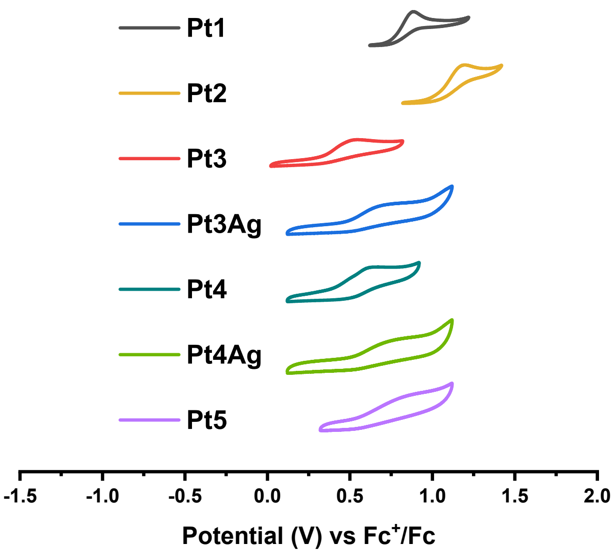

Figure S4. Cyclic voltammograms of all studied Pt(II) complexes at RT in CH_2CH_2 for oxidation potential.

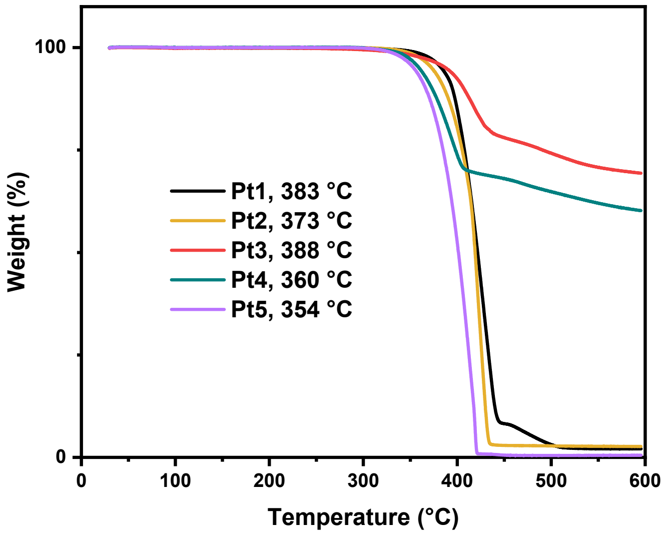

Figure S5. Thermogravimetric data of studied Pt(II) complexes with decomposition temperature (T_d) at a weight loss of 5 wt%.

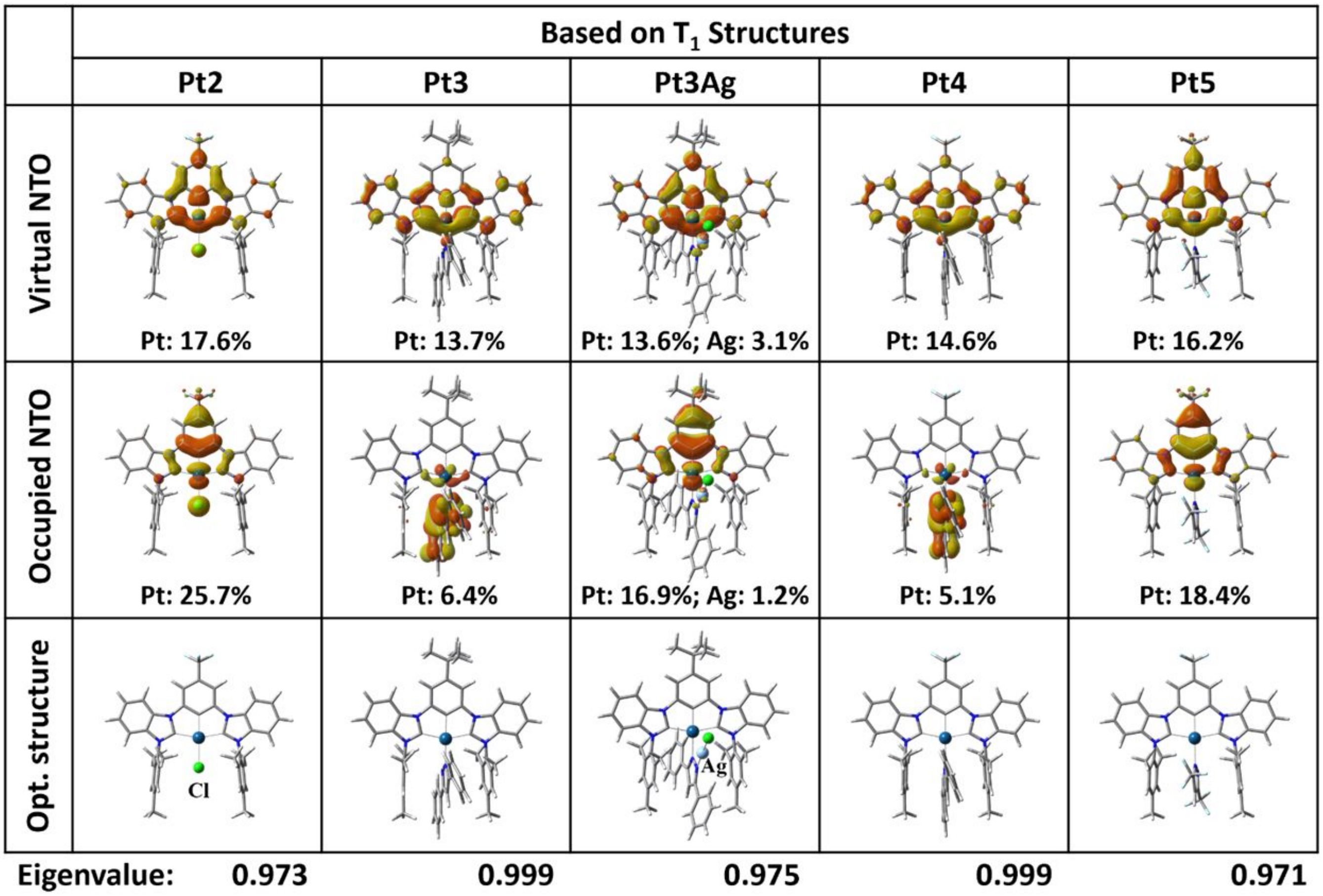

Figure S6. Dominant eigenvalues and NTO pairs for $S_0 \rightarrow T_1$ excitation of the studied complexes at their optimized T_1 structures in toluene, including the contributions of the Pt(II) center and Ag atom to the NTOs.

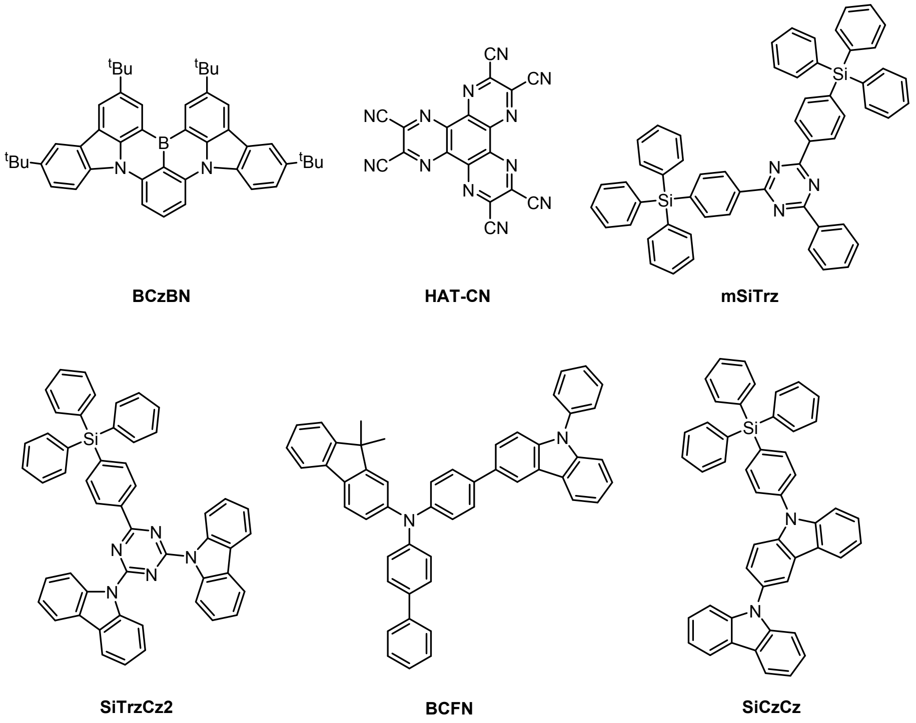

Figure S7. Structure of the employed materials in the doped OLED devices.

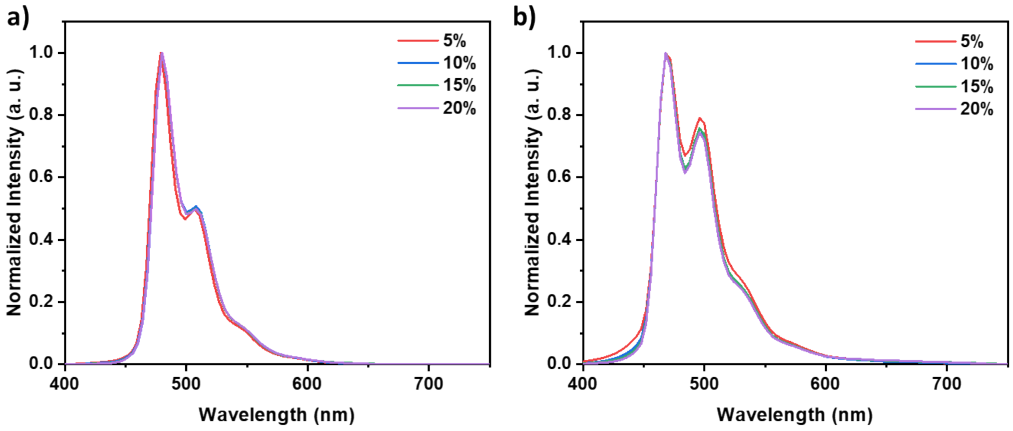

Figure S8. Electroluminescence spectra of PhOLEDs with different doping concentration of a) **Pt2** and b) **Pt5**.

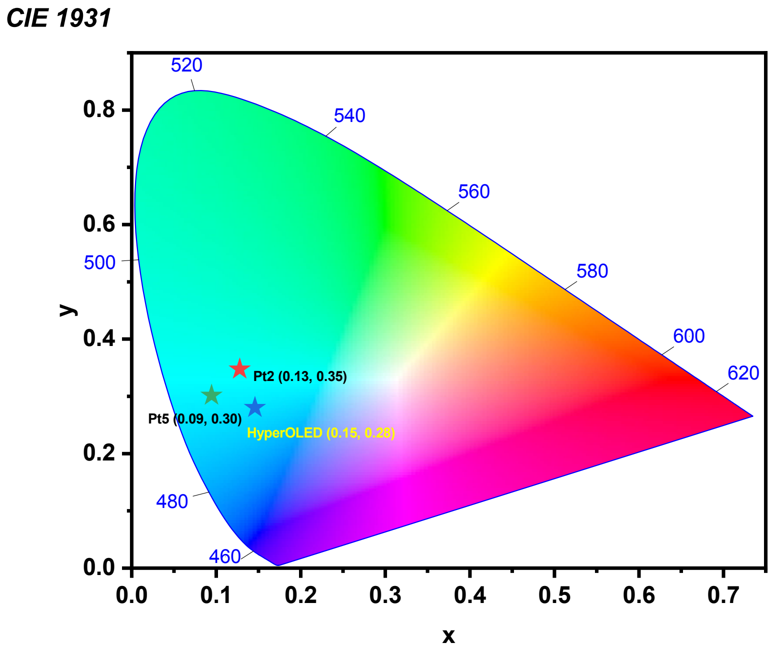

Figure S9. $CIE_{x,y}$ coordinates for phosphors **Pt2** and **Pt5** and corresponding hyper-OLED devices employing terminal emitter **BCzBN**.

Table S1. Summarized photophysical data of the studied Pt(II) complexes.

	λ_{PL} [nm] [a]	FWHM	PLQY [a]	τ_{obs} [μs]	τ_{rad} [μs]	k_r [10^5 s^{-1}] [b]	k_{nr} [10^5 s^{-1}] [b]
**Pt1**	495, 529, 567	52 nm	0.82	2.31	2.82	3.55	0.78
**Pt2**	476, 506, 540	40 nm	0.82	2.08	2.54	3.94	0.87
**Pt3**	486, 518, 554	54 nm	0.58	1.87	3.22	3.10	2.25
**Pt4**	499, 523	82 nm	0.27	1.59	5.89	1.70	4.59
**Pt5**	462, 492, 524	45 nm	0.70	3.26	4.66	2.15	0.92

[a] Photophysical data recorded in drop-cast neat film at RT. PLQY was recorded using Edinburgh FLS 980 system with integration sphere.

[b] k_r and k_{nr} represent radiative and nonradiative rate constant.

Table S2. Summarized electrochemical data of the studied Pt(II) complexes.

	E^{ox}_{onset} (V) [a]	E_{HOMO} (eV)	abs_{onset} (nm)	E_g [b] (eV)	E_{LUMO} [b] (eV)
**Pt1**	0.75	−5.55	448	2.77	−2.78
**Pt2**	1.02	−5.82	436	2.84	−2.98
**Pt3**	0.34	−5.14	445	2.79	−2.35
**Pt3Ag**	0.48	−5.28	442	2.81	−2.47
**Pt4**	0.40	−5.20	429	2.89	−2.31
**Pt4Ag**	0.51	−5.31	426	2.91	−2.40
**Pt5**	0.56	−5.36	409	3.03	−2.33

[a] E^{ox}_{onset} (V) refers to the onset of oxidation peak, referenced to ferrocene standard (Fc/Fc^+ = 0.38 V) in CH_2Cl_2 solution at RT.

[b] $E_{HOMO} = -(E_{ox} + 4.8)$ eV, $E_g = 1240/\lambda_{onset}$, and $E_{LUMO} = E_{HOMO} + E_g$.

Table S3. Computed Pt-ligand bond distances (Å) of the studied Pt(II) complexes at their optimized S_0 (normal font) and T_1 (italic and bold font in parentheses) structures in toluene at B3LYP-D3(BJ)/def2-SVP level.

	**Pt-C$_{(Ph)}$**	**Pt-C$_{(carbene)}$**	**Pt-Cl or Pt-N$_{(pz)}$**
**Pt2**	1.955 (***1.911***)	2.040/2.041 (***2.037/2.037***)	2.431 (***2.424***)
**Pt3**	1.966 (***1.972***)	2.044/2.045 (***2.020/2.017***)	2.128 (***2.112***)
**Pt3Ag**	1.962 (***1.909***)	2.047/2.061 (***2.027/2.041***)	2.145 (***2.157***)
**Pt4**	1.965 (***1.968***)	2.040/2.045 (***2.017/2.018***)	2.131 (***2.118***)
**Pt5**	1.963 (***1.909***)	2.050/2.052 (***2.038/2.039***)	2.152 (***2.165***)

Table S4. Performance of phosphorescent and hyper-OLED devices based on **Pt2** and **Pt5** phosphors and **BCzBN** as terminal emitter.

Emitter	λ_{max}[a] (nm)	FWHM	V_{on}[b] (V)	CIE[c] (x,y)	L_{max} (cd m^{-2})	CE (cd A^{-1})	PE (lm W^{-1})	EQE[d] (%)
**5 wt% Pt2**	480; 508; 543	27	2.8	0.128, 0.363	50,330	37.3 / 36.3 / 30.6	31.3 / 26.8 / 15.4	18.8 / 18.3 / 15.6
**15 wt% Pt2**	480; 508; 543	27	2.7	0.128, 0.375	94,660	38.6 / 37.6 / 31.6	41.4 / 29.0 / 14.4	19.1 / 18.7 / 15.9
**20 wt% Pt2**	480; 508; 543	27	2.7	0.128, 0.377	92,480	35.8 / 35.6 / 31.0	34.8 / 24.8 / 14.7	17.6 / 15.7 / 15.5
**5 wt% Pt5**	468; 496; 530	49	2.8	0.141, 0.277	19,280	31.6 / 29.0 / 19.6	35.1 / 20.7 / 9.4	17.5 / 16.3 / 11.1
**15wt% Pt5**	468; 496; 530	49	2.7	0.141, 0.279	16,810	32.9 / 25.7 / 12.1	36.7 / 18.0 / 4.6	18.1 / 14.3 / 6.9
**20 wt% Pt5**	468; 496; 530	49	2.7	0.141, 0.278	15,630	29.8 / 22.8 / 11.3	32.0 / 15.9 / 4.5	16.1 / 12.7 / 6.4
**Hyper-OLEDs with 2 wt% BCzBN**								
**10 wt% Pt2**	484	27	2.7	0.084, 0.370	98,710	38.8 / 31.6 / 23.9	42.8 / 22.0 / 11.0	22.2 / 18.2 / 14.1
**10 wt% Pt5**	484	29	2.7	0.087, 0.344	29,870	34.5 / 27.2 / 19.4	38.6 / 19.5 / 9.3	20.8 / 16.4 / 11.9

[a] EL peak max. recorded at the luminance of 1000 cd m^{-2} and FWHM indicated the full width at half maxima.

[b] Turn-on voltage at 1 cd m^{-2}.

[c] CIE coordinates at the luminance of 1000 cd m^{-2}.

[d] The maximum value, and value recorded at 1000 cd m^{-2} and 10000 cd m^{-2}.

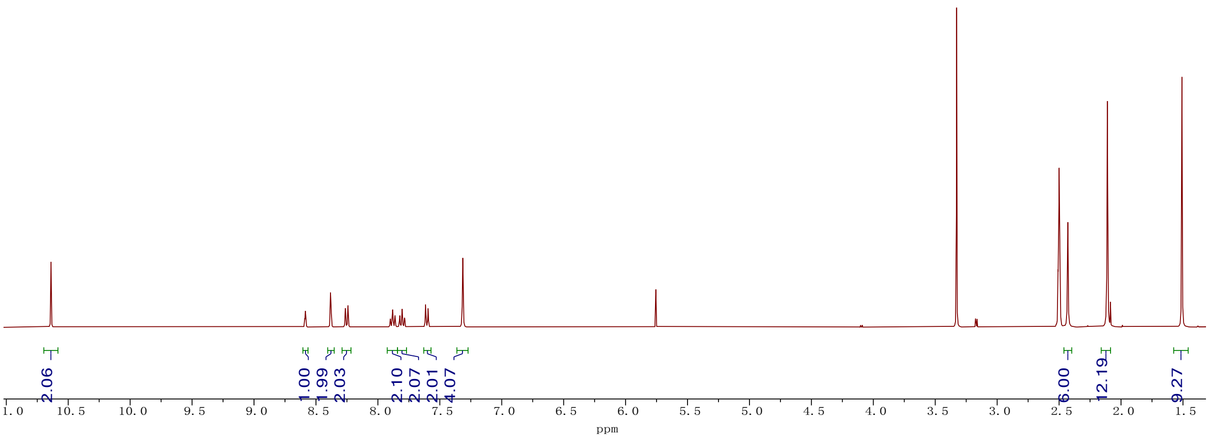

Figure S10. ^{1}H NMR (400 MHz) spectrum of **C1** recorded in DMSO-d_6 at RT.

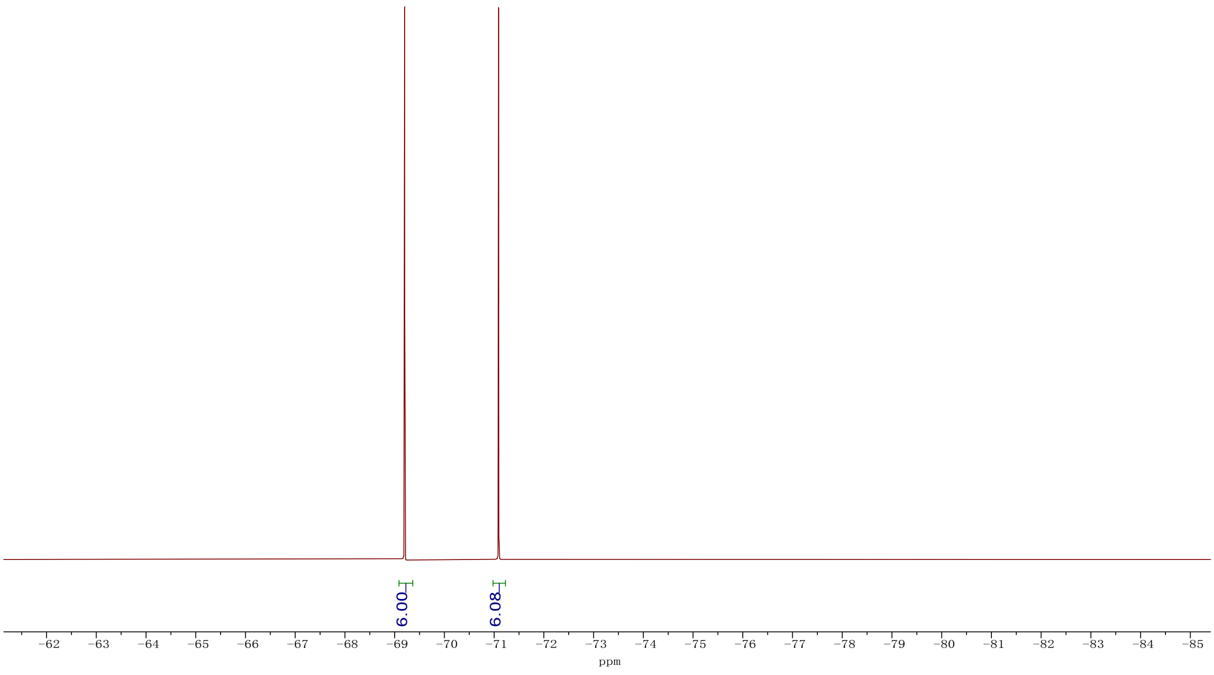

Figure S11. ^{19}F NMR (376 MHz) spectrum of **C1** recorded in DMSO-d_6 at RT.

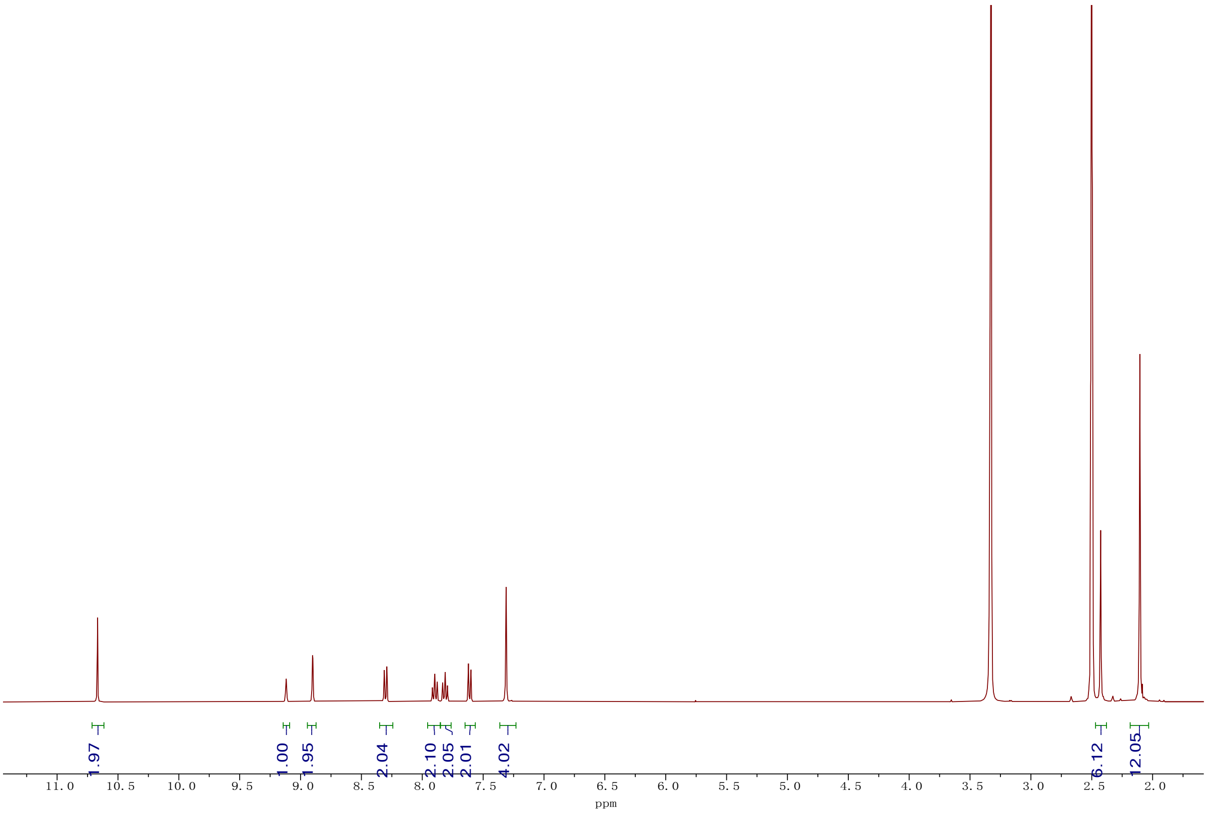

Figure S12. ^{1}H NMR (400 MHz) spectrum of **C2** recorded in DMSO-d_6 at RT.

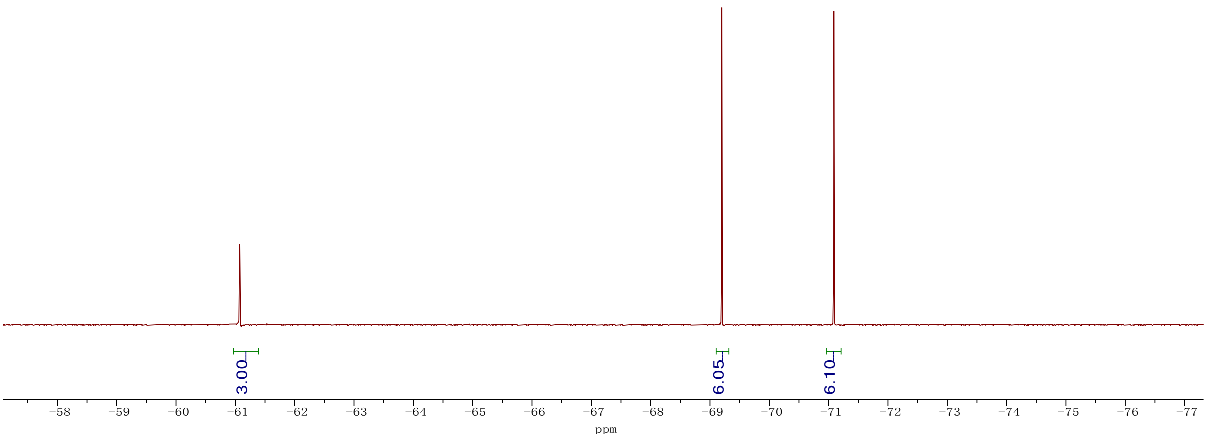

Figure S13. ^{19}F NMR (376 MHz) spectrum of **C2** recorded in DMSO-d_6 at RT.

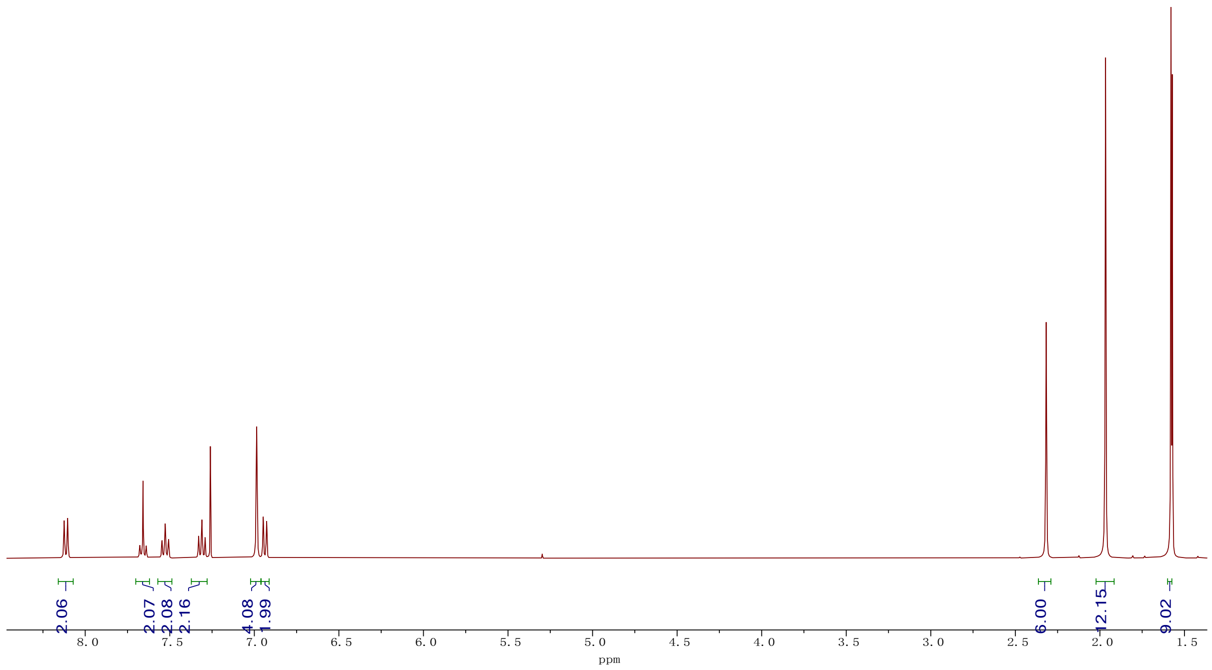

Figure S14. ^{1}H NMR (400 MHz) spectrum of **Pt1** recorded in $CDCl_3$ at RT.

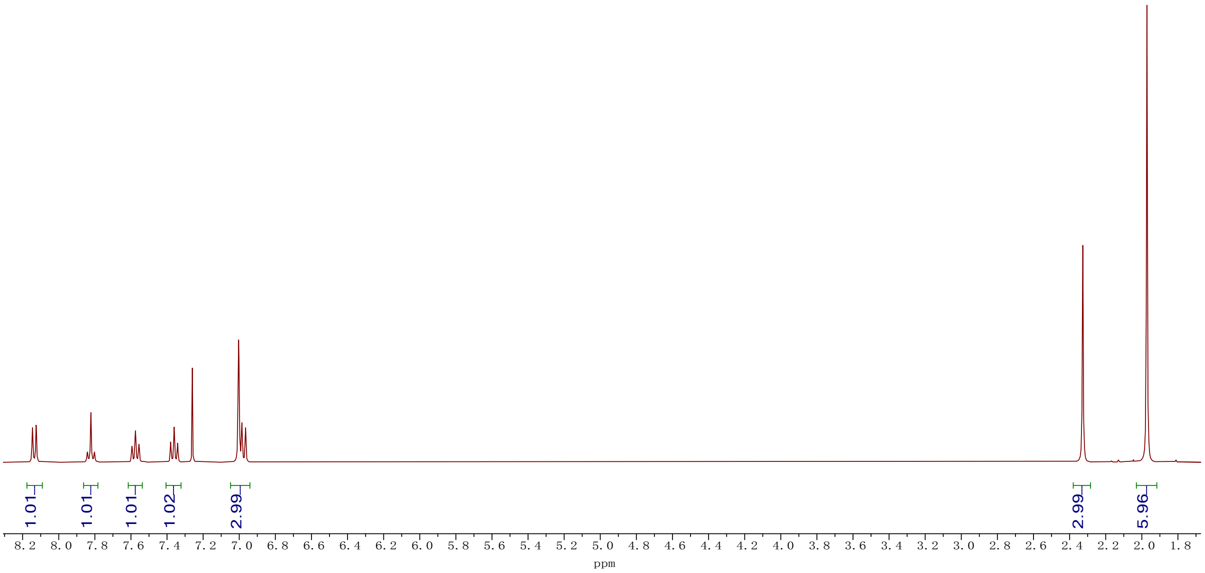

Figure S15. ^{1}H NMR (400 MHz) spectrum of **Pt2** recorded in $CDCl_3$ at RT.

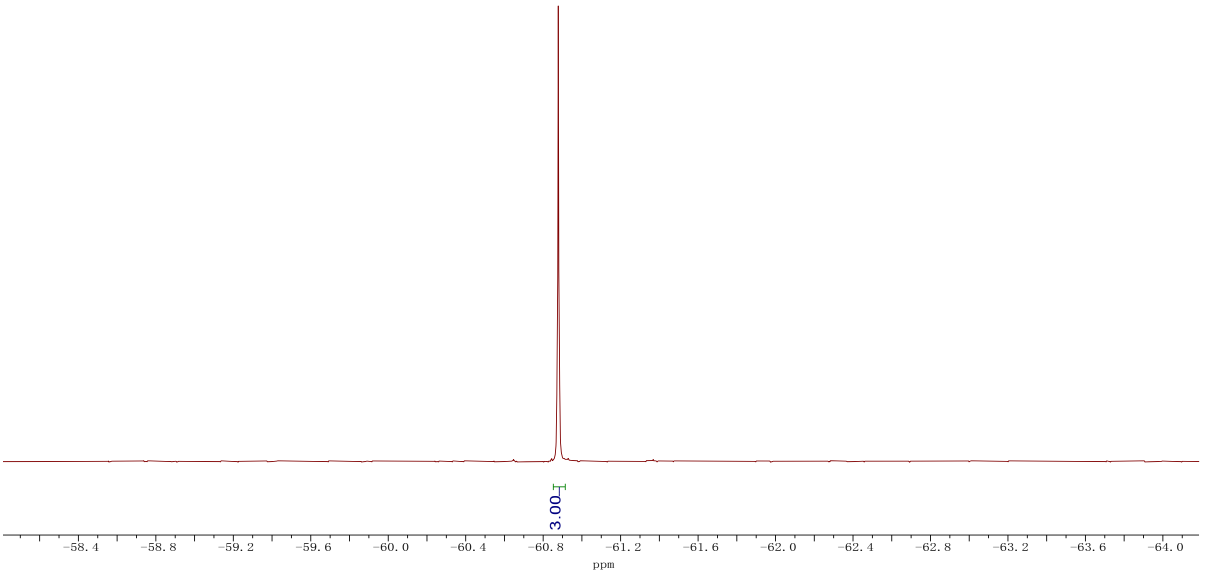

Figure S16. ^{19}F NMR (376 MHz) spectrum of **Pt2** recorded in $CDCl_3$ at RT.

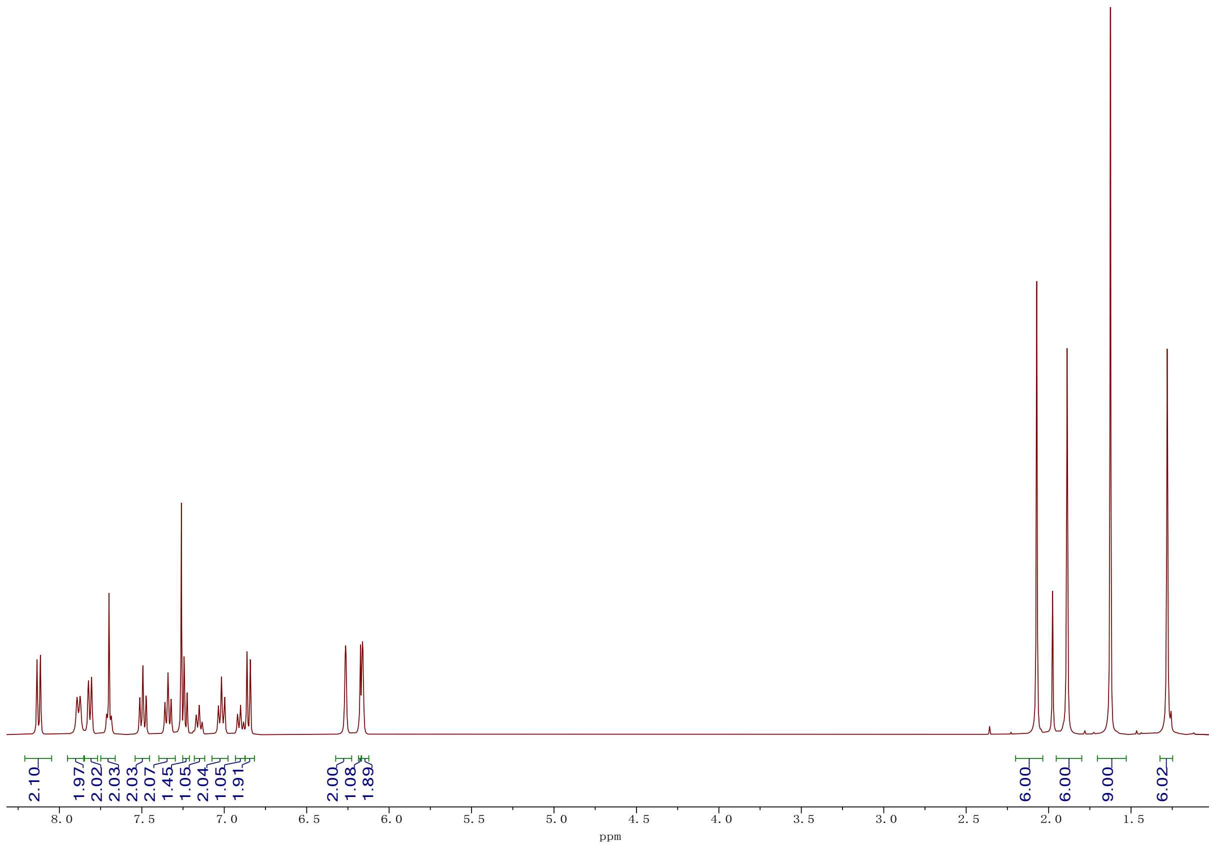

Figure S17. ^1H NMR (400 MHz) spectrum of **Pt3** recorded in $CDCl_3$ at RT.

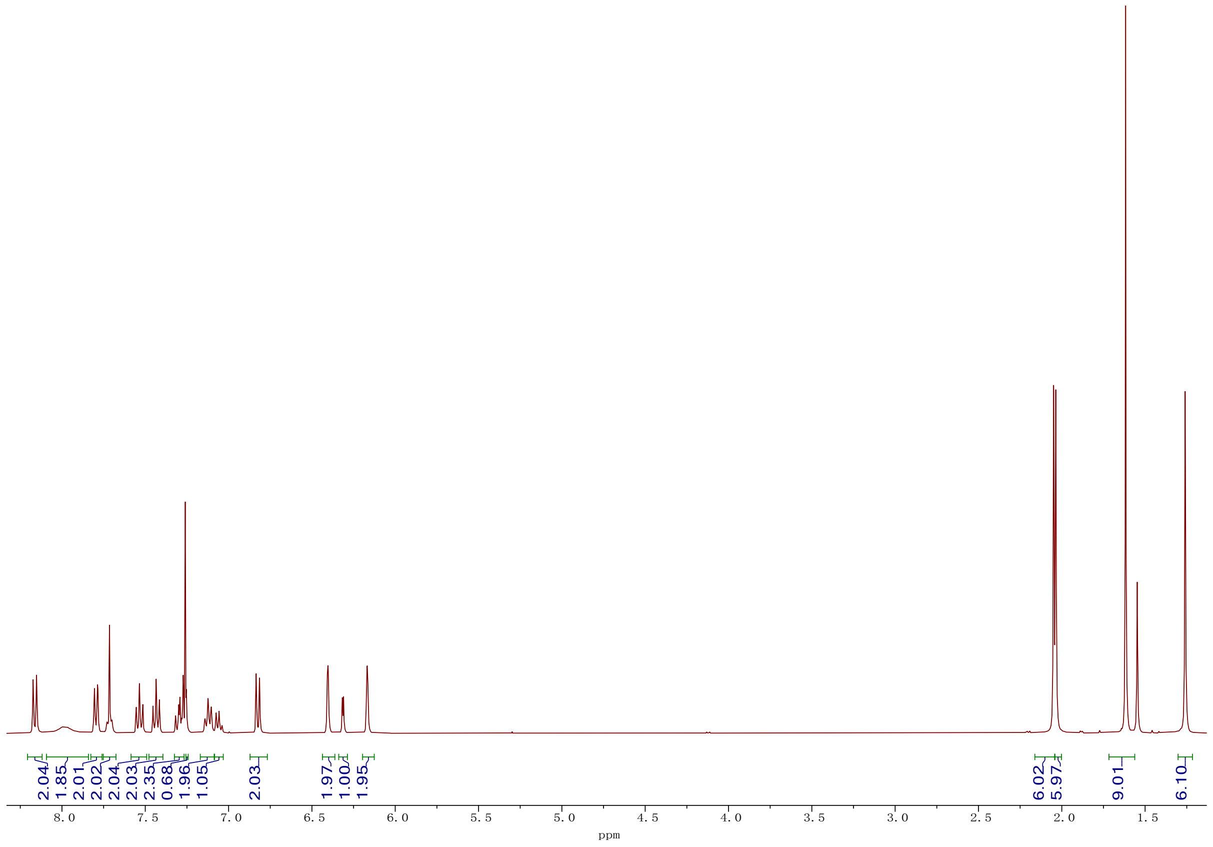

Figure S18. ^1H NMR (400 MHz) spectrum of **Pt3Ag** recorded in $CDCl_3$ at RT.

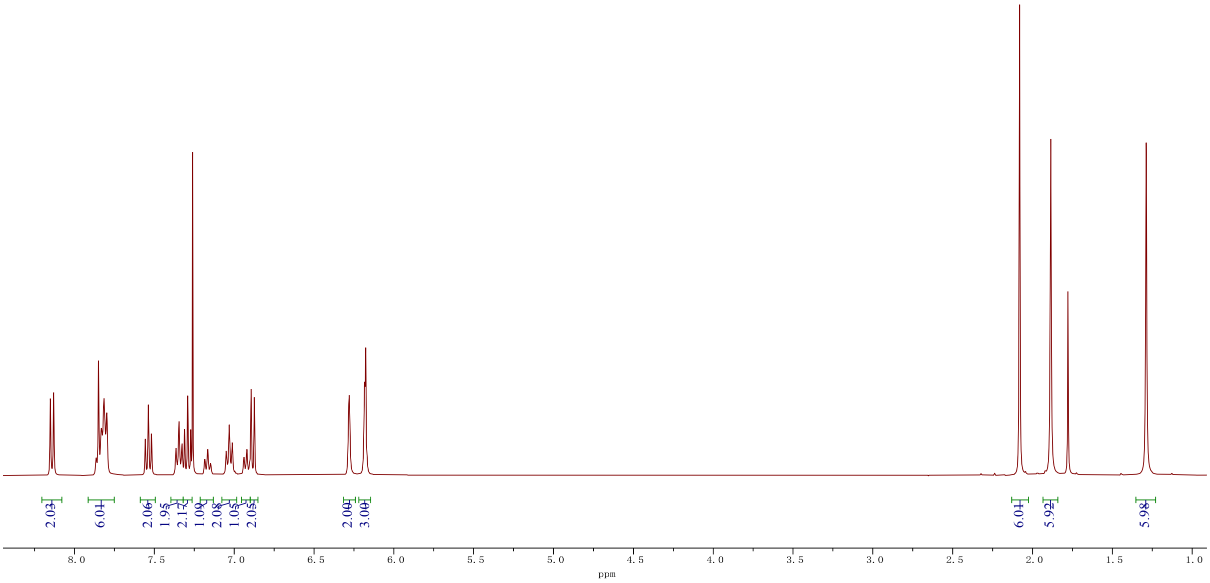

Figure S19. ^{1}H NMR (400 MHz) spectrum of **Pt4** recorded in $CDCl_3$ at RT.

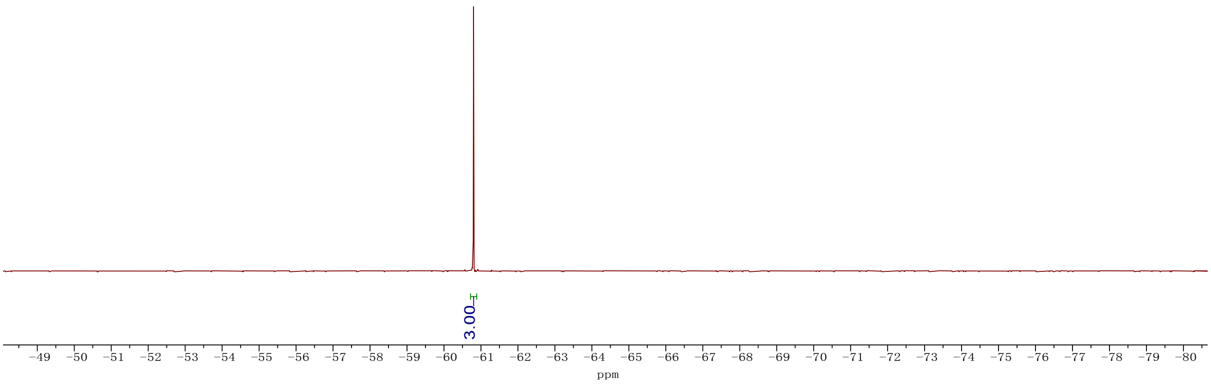

Figure S20. ^{19}F NMR (376 MHz) spectrum of **Pt4** recorded in $CDCl_3$ at RT.

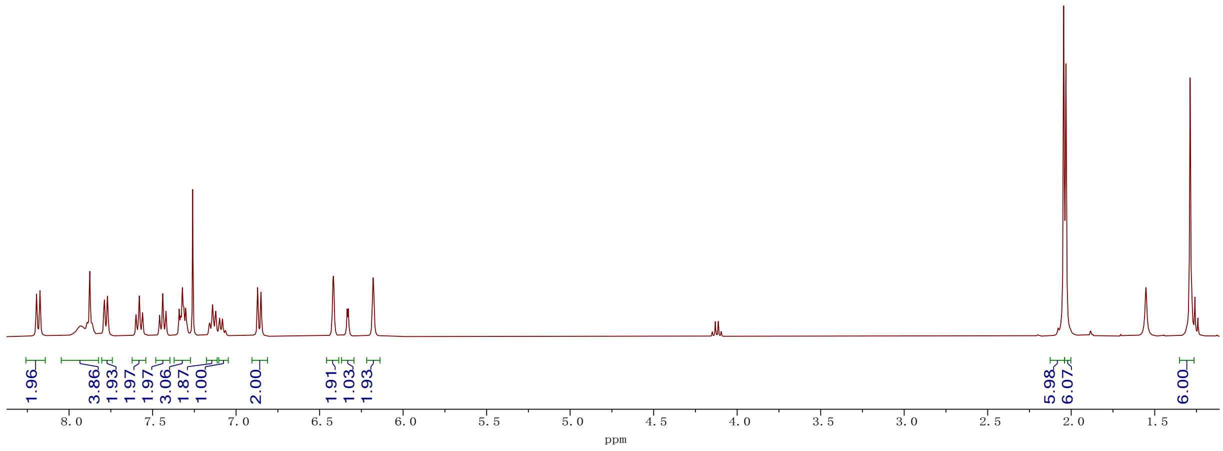

Figure S21. ^{1}H NMR (400 MHz) spectrum of **Pt4Ag** recorded in $CDCl_3$ at RT.

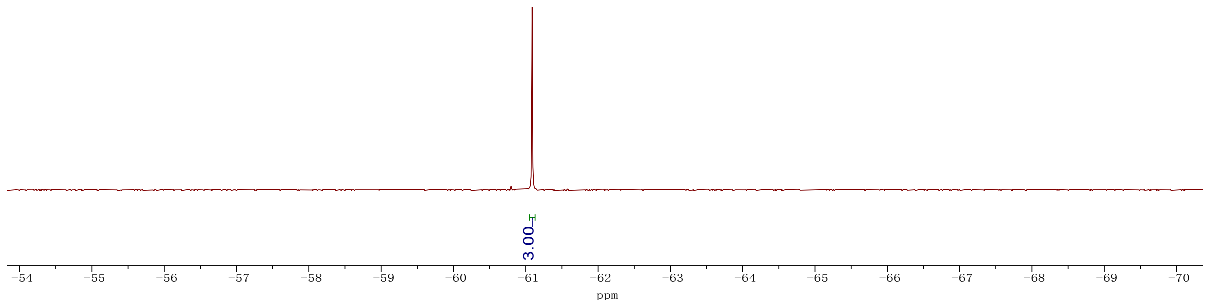

Figure S22. ^{19}F NMR (376 MHz) spectrum of **Pt4Ag** recorded in $CDCl_3$ at RT.

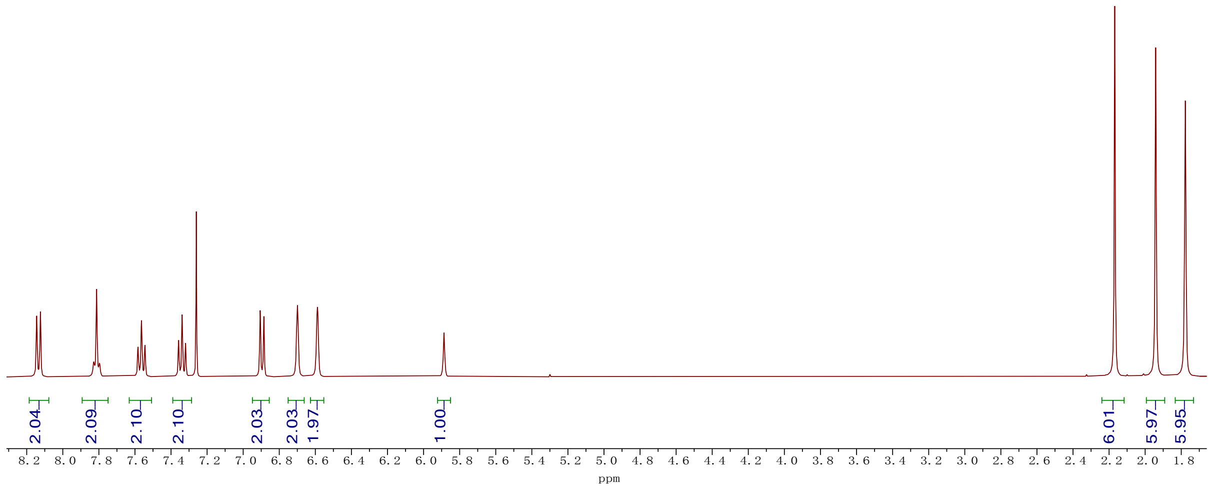

Figure S23. ^{1}H NMR (400 MHz) spectrum of **Pt5** recorded in $CDCl_3$ at RT.

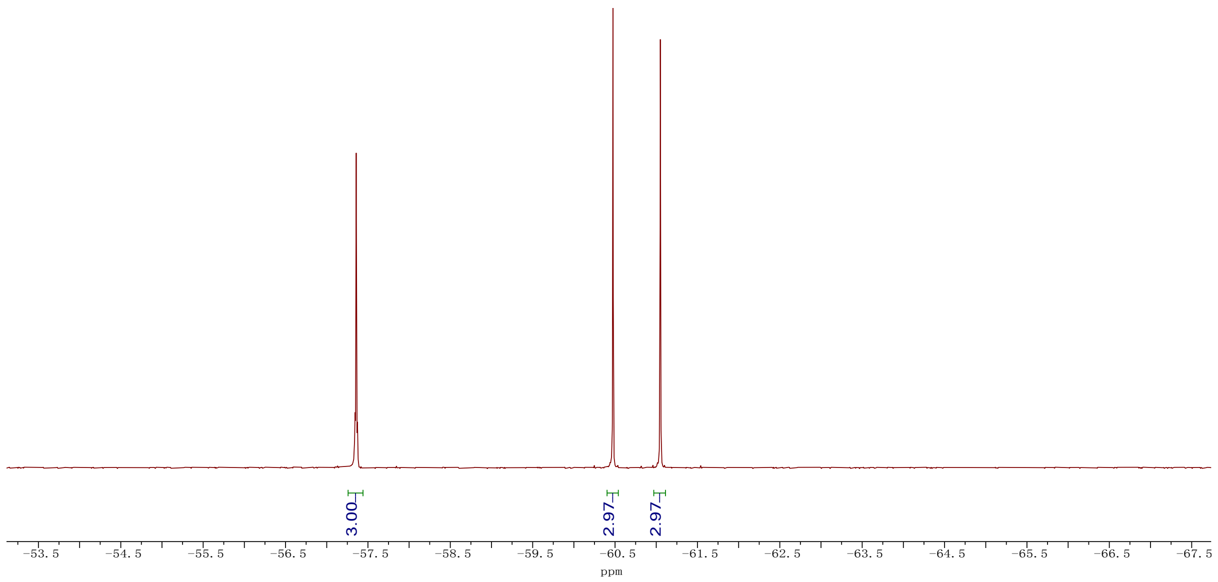

Figure S24. ^{19}F NMR (376 MHz) spectrum of **Pt5** recorded in $CDCl_3$ at RT.

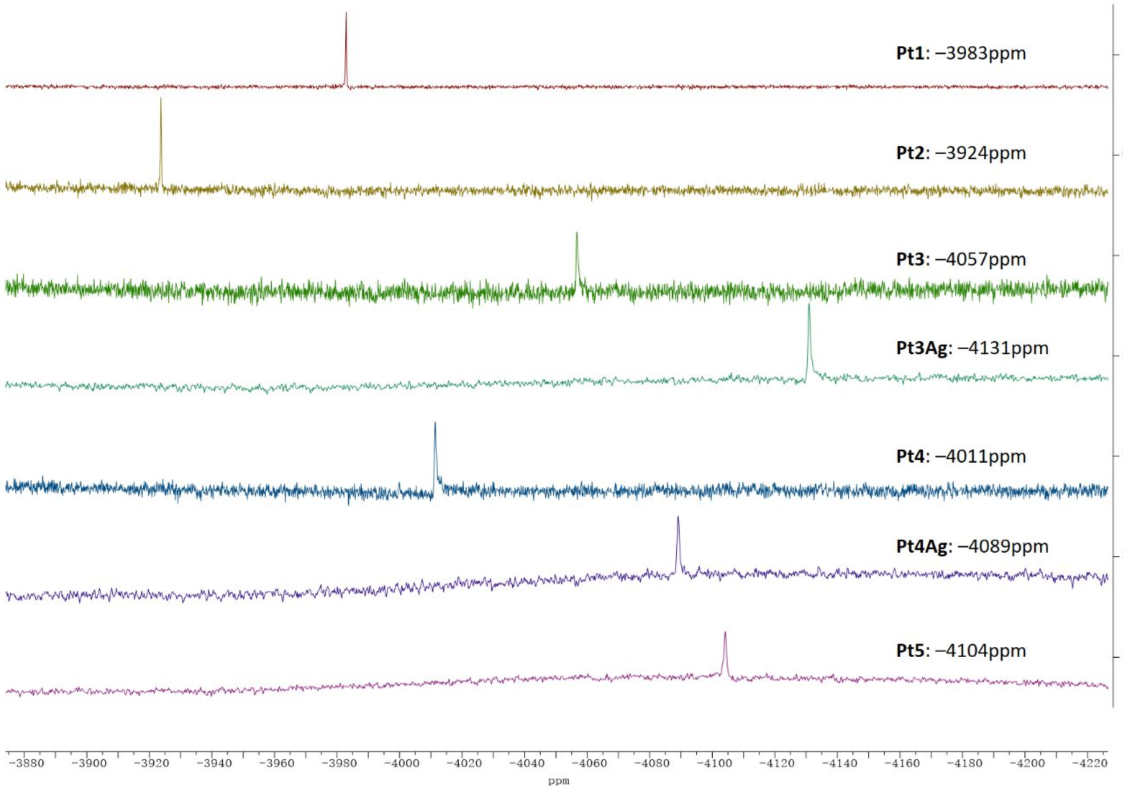

Figure S25. ^{195}Pt NMR (86 MHz) spectrum of studied Pt(II) complexes recorded in $CDCl_3$ at RT.

References

(1) Lee, C.; Yang, W.; Parr, R. G. Development of the Colle-Salvetti correlation-energy formula into a functional of the electron density. *Phys. Rev. B* **1988**, *37* (2), 785-789.

(2) Becke, A. D. Density-functional thermochemistry. III. The role of exact exchange. *J. Chem. Phys.* **1993**, *98* (7), 5648-5652.

(3) Weigend, F.; Ahlrichs, R. Balanced basis sets of split valence, triple zeta valence and quadruple zeta valence quality for H to Rn: Design and assessment of accuracy. *Phys. Chem. Chem. Phys.* **2005**, *7* (18), 3297-3305, 10.1039/B508541A. DOI: 10.1039/B508541A.

(4) Weigend, F. Accurate Coulomb-fitting basis sets for H to Rn. *Phys. Chem. Chem. Phys.* **2006**, *8* (9), 1057-1065, 10.1039/B515623H. DOI: 10.1039/B515623H.

(5) Grimme, S.; Ehrlich, S.; Goerigk, L. Effect of the damping function in dispersion corrected density functional theory. *J. Comput. Chem.* **2011**, *32* (7), 1456-1465. DOI: 10.1002/jcc.21759.

(6) Frisch, M. J.; Trucks, G. W.; Schlegel, H. B.; Scuseria, G. E.; Robb, M. A.; Cheeseman, J. R.; Scalmani, G.; Barone, V.; Mennucci, B.; Petersson, G. A.; et al. Gaussian 16, Revision C.01. *Gaussian 16, Revision C.01; Gaussian Inc.* **2016**, Wallingford, CT.

(7) Miertuš, S.; Scrocco, E.; Tomasi, J. Electrostatic interaction of a solute with a continuum. A direct utilizaion of AB initio molecular potentials for the prevision of solvent effects. *Chem. Phys.* **1981**, *55* (1), 117-129.

(8) Miertuŝ, S.; Tomasi, J. Approximate evaluations of the electrostatic free energy and internal energy changes in solution processes. *Chem. Phys.* **1982**, *65* (2), 239-245.

(9) Adamo, C.; Jacquemin, D. The calculations of excited-state properties with Time-Dependent Density Functional Theory. *Chem. Soc. Rev.* **2013**, *42* (3), 845-856, 10.1039/C2CS35394F. DOI: 10.1039/C2CS35394F.

(10) Laurent, A. D.; Adamo, C.; Jacquemin, D. Dye chemistry with time-dependent density functional theory. *Phys. Chem. Chem. Phys.* **2014**, *16* (28), 14334-14356, 10.1039/C3CP55336A. DOI: 10.1039/C3CP55336A.

(11) Martin, R. L. Natural Transition Orbitals. *J. Chem. Phys.* **2003**, *118* (11), 4775-4777. DOI: 10.1063/1.1558471.

(12) Hirshfeld, F. L. Bonded-atom fragments for describing molecular charge densities. *Theo. Chim. Acta* **1977**, *44* (2), 129-138. DOI: 10.1007/BF00549096.

(13) Lu, T.; Chen, F. Multiwfn: A multifunctional wavefunction analyzer. *J. Comput. Chem.* **2012**, *33* (5), 580-592. DOI: https://doi.org/10.1002/jcc.22885 (acccessed 2026/04/08).

(14) Lu, T. A comprehensive electron wavefunction analysis toolbox for chemists, Multiwfn. *J. Chem. Phys.* **2024**, *161* (8), 082503. DOI: 10.1063/5.0216272.

(15) de Souza, B.; Farias, G.; Neese, F.; Izsák, R. Predicting Phosphorescence Rates of Light Organic Molecules Using Time-Dependent Density Functional Theory and the Path Integral Approach to Dynamics. *J. Chem. Theory Comput.* **2019**, *15* (3), 1896-1904. DOI: 10.1021/acs.jctc.8b00841.

(16) Neese, F.; Wennmohs, F.; Becker, U.; Riplinger, C. The ORCA quantum chemistry program package. *J. Chem. Phys.* **2020**, *152* (22), 224108. DOI: 10.1063/5.0004608 (acccessed 5/2/2023).

(17) Neese, F. Software update: The ORCA program system - Version 5.0. *WIREs Comput. Mol.*

Sci. **2022**, *12* (5), e1606. DOI: https://doi.org/10.1002/wcms.1606.
(18) van Lenthe, E.; Baerends, E. J.; Snijders, J. G. Relativistic regular two - component Hamiltonians. *J. Chem. Phys.* **1993**, *99* (6), 4597-4610. DOI: 10.1063/1.466059 (acccessed 5/2/2023).
(19) van Lenthe, E.; Baerends, E. J.; Snijders, J. G. Relativistic total energy using regular approximations. *J. Chem. Phys.* **1994**, *101* (11), 9783-9792. DOI: 10.1063/1.467943.
(20) Pye, C. C.; Ziegler, T. An implementation of the conductor-like screening model of solvation within the Amsterdam density functional package. *Theor. Chem. Acc.* **1999**, *101* (6), 396-408, journal article. DOI: 10.1007/s002140050457.
